# Multicenter Bonding in Wilkinson’s Catalyst: A Theoretical Study from a Distorted Monomer to a Localized Dimer

**DOI:** 10.1021/acs.jpca.6c04219

**Published:** 2026-09-28

**Authors:** Aleksandr Zaichenko, Holger F. Bettinger, Ivana Fleischer, Doreen Mollenhauer

**Affiliations:** † Helmholtz Institute for Polymers in Energy Applications Jena (HIPOLE Jena), Lessingstrasse 12-14, Jena 07743, Germany; ‡ Helmholtz-Zentrum Berlin für Materialien Und Energie GmbH (HZB), Hahn-Meitner-Platz 1, Berlin 14109, Germany; § Institute for Technical and Environmental Chemistry, Friedrich Schiller University Jena, Philosophenweg 7a, Jena 07743, Germany; ∥ 9188 Institut für Organische Chemie, Auf der Morgenstelle 18, Tübingen 72076, Germany

## Abstract

In this work, we present a computational study of Wilkinson’s catalyst, [Rh­(PPh_3_)_3_Cl], focusing on the connection between geometric structure, electronic structure, and thermodynamics in its monomeric and dimeric forms, as well as the coordination of hydrogen atoms. Scalar-relativistic DFT with modern meta-GGA-based functionals, coupled-cluster benchmarks, and bonding analyses (NBO/NRT) show that steric effects and noncovalent interactions control deviations from ideal square-planar geometry in the metastable monomeric structure as well as affect dissociation and dimerization energetics, whereas electronic factors dominate the dimer structure and enthalpy. The monomer is characterized by intrinsically delocalized multicenter bonding, best described as two crossed three-center, four-electron (3c–4e) interactions spanning Rh, while entropy-controlled dimerization induces a qualitatively different and more localized bonding regime. In both species, Rh acts as an electron acceptor in donor-acceptor Rh–ligand interactions and remains consistent with a 16-electron configuration. Thermodynamic results support the experimentally observed tendency toward dimerization in solution, highlighting the importance of multicenter bonding, entropic factors, and steric control for interpreting the structure and reactivity of Wilkinson’s catalyst.

## Graphical abstract

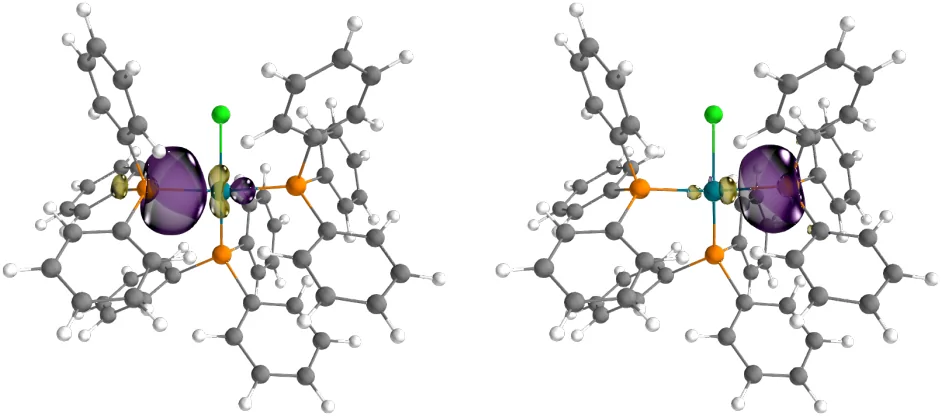

## Introduction

Wilkinson’s catalyst, [Rh­(PPh_3_)_3_Cl], is a transition-metal complex that occupies a central position in preparative transition-metal coordination chemistry and homogeneous catalysis.^1–4^ This catalyst has a broad range of applications, such as hydrogenation,^5^ hydroformylation,^6^ hydroboration,^7^ hydrosilylation,^8^ decarbonylation,^9^ and the catalysis of (forbidden) pericyclic reactions.^10–12^ The characteristic electronic structure of such complexes is usually that of a d^8^ Rh­(I) center, which commonly gives rise to a (distorted) square-planar coordination geometry. Wilkinson’s catalyst is a four-coordinate, distorted square-planar complex and is commonly formulated as a 16-electron species consistent with the 16-electron rule. This d^8^/16-electron electronic configuration leads to well-defined geometries and reactivity patterns that have been extensively explored both experimentally and theoretically.^1,13^

Despite this apparent simplicity, the nature of metal-ligand bonding in square-planar environments may be distorted by ligands and may involve significant electron delocalization and donor-acceptor interactions that are not always adequately captured by classical two-center bonding models. Wilkinson’s catalyst, bearing bulky ligands, has a tendency to dimerize, as described in the literature,^5,14^ while the underlying bonding patterns remain less clearly understood. The bulky ligands induce deviations from idealized planar complex geometries, as evidenced by crystallographic studies.^15^ Accordingly, the study of bonding concepts and energetics in planar and distorted ligand environments, as well as bonding with hydrogen atoms, is of interest for understanding bonding, dimerization and stabilization in these systems.

From a theoretical perspective, a reliable analysis of bonding in square-planar complexes requires a balanced treatment of electron correlation and relativistic effects, especially for systems containing heavier 4d elements such as rhodium (Rh). Modern density functional methods, complemented by wavefunction-based approaches and real-space bonding analyses, offer a framework to address these questions in a systematic and methodologically robust manner.

In this work, we present a comprehensive computational study of Wilkinson’s catalyst, focusing on the relationship between geometric and electronic structure, thermodynamics, and the nature of the Rh­(I) coordination sphere in both monomeric and dimeric forms. Using scalar-relativistic density functional theory (DFT), coupled-cluster benchmark calculations, and complementary bonding analyses based on natural bond orbitals (NBO), natural resonance theory (NRT), and electron-density-based methodologies, we demonstrate that the monomeric complex is characterized by intrinsically delocalized three-center, four-electron interactions spanning the Rh center that are energetically preferred in comparison to localized two-center bonds. The quantum theory of atoms and molecules (QTAIM) and non-covalent interaction analysis were applied to analyze the coordination spheres of the Rh atom, providing powerful methods of theoretical metal complex chemistry, as demonstrated in the literature.^16–18^ In contrast, dimerization leads to a qualitatively different, predominantly localized bonding regime and purely entropic reaction control. The present results provide a coherent electronic-structure interpretation of the bonding and reactivity of Wilkinson’s catalyst and highlight the importance of multicenter bonding concepts and steric factors for understanding its chemical behavior.

## Computational Details

All DFT calculations were performed using the TURBOMOLE^19,20^ program package version 7.9.0. Structure optimizations and vibrational frequency calculations for all considered systems were carried out at the DFT level using the resolution of identity RI-JK approximation for the Coulomb (J) and exchange (K) terms^21^ with the TPSSh^22,23^ hybrid meta-GGA functional in combination with Grimme’s D4 ^24^ dispersion correction. Harmonic vibrational analyses confirmed that the optimized structures correspond to true minima on the potential energy surface (convergence criteria: 10^–6^ Ha), apart from a few low-frequency imaginary modes associated with hindered internal rotations in symmetric structures (<10 cm^–1^ for the dimer). Infrared (IR) spectra were obtained within the harmonic approximation with major isotope masses. Reaction energies were corrected for basis set superposition error (BSSE) using the counterpoise method. Additional global structure analysis including conformer searches, were performed using xTB-CREST software.^25^

For Rh, an effective core potential (ECP) approach was employed using the ECP-def2-TZVPP basis set in conjunction with the corresponding def2-ECP (ECP28MDF), replacing 28 core electrons and leaving 17 electrons treated explicitly, while all remaining ligand atoms were described using the def2-SVP basis set.^26–28^ For the study of H_2_ coordination, the def2-TZVP basis set was used for H atoms coordinated to the Rh center. Solvent effects were included during structure optimizations and frequency calculations using the conductor like screening model COSMO,^29–31^ an implicit solvation model, with toluene as the solvent (dielectric constant ε = 2.38). For all DFT calculations the m5 numerical integration grid was utilized. Test calculations employing the full triple-zeta def2-TZVP basis set did not reveal any significant structural differences in the monomer.

Scalar relativistic effects were treated using the exact two-component (X2C)^32^ formalism without the RI approximation. Fully scalar relativistic all-electron single-point calculations were performed using the x2c-def2-TZVPPall basis set for the Rh atom and x2c-def2-TZVPall for the remaining atoms.^33^ These relativistic calculations, as well as all wavefunction-based calculations, were carried out in the gas phase using structures optimized at the TPSSh-D4/[def2-SVP (non-Rh) + def2-TZVPP/def2-ECP (Rh)] (COSMO, toluene) level and were used exclusively for electronic-structure and electron-density analyses. For methodological comparison, additional X2C-based DFT calculations were performed using the r^2^SCAN^34^ functional with identical basis set combinations and numerical settings.

To enable a reliable analysis of the isolated complex core, to isolate metal-ligand coordination effects from bulky-ligand effects, and to make correlated CCSD calculations feasible while retaining the relevant coordination environment of Rh, an additional distorted square-planar model (DSPC) was constructed. In this structure, the coordination geometry around Rh was kept fixed to that of the Wilkinson’s monomer, while only the hydrogen substituents on the ligands (replacing the phenyl groups) were allowed to relax. This strategy minimizes phenyl-ligand effects without changing the lengths and angles of coordination bonds.

To benchmark the DFT results, wavefunction-based coupled-cluster calculations at the X2C-CCSD^35^ level were carried out for reduced model systems. These calculations employed the x2c-def2-TZVPPall basis set for Rh and x2c-def2-SVP for the remaining atoms and were performed using the CFOUR program package.^36–43^

Molecular orbitals were converted from MOLDEN format using Molden2AIM^44^ for subsequent Natural Bond Orbital (NBO) analyses. The topology of the electron density was analyzed within the Quantum Theory of Atoms in Molecules (QTAIM)^45,46^ framework using the Multiwfn program.^47,48^ Bond critical points and related topological descriptors were identified to characterize metal-ligand bond localization.

Buried- and free-volumes descriptors of the molecules were calculated using the SambVca 2.1 software with the default setting of atomic radii.^49^

Chemical bonding was investigated using NBO 7,^50–52^ including Natural Population Analysis (NPA), Mayer bond orders (MBO), Wiberg bond indices (WBI), Natural Resonance Theory (NRT),^53–55^ Natural Bond Orbital (NBO) analysis, hyperbonding analysis,^52^ and Natural Localized Molecular Orbitals (NLMOs). Molecular structures, molecular orbitals, and electron-density-related properties were visualized using Pegamoid,^56^ Chemcraft,^57^ and LaTeX-based tools for the preparation of publication-quality figures. IR spectra were constructed using MOLDEN.^58^ All visualized orbitals are presented with an isosurface value of 0.05. For visualization of the non-covalent interaction analysis results, aPyVista based script developed in our group was used.

## Results and Discussion

### Geometric Structures of the Monomeric and Dimeric Complexes

The optimized solution-phase structure of the monomeric Wilkinson’s catalyst, [Rh­(PPh_3_)_3_Cl], deviates noticeably from an ideal square-planar coordination geometry around Rh, as reflected by the dihedral angle *φ*_Rh‑P1‑P3‑Cl_ (Figures 1 and 2, Table 1), which would be 0° for a perfectly planar arrangement. This deviation originates from pronounced steric congestion caused by the bulky phenyl substituents of PPh_3_ and is consistent with the deformation patterns reported in the literature based on X-ray crystallographic data: for trans-oriented ligands, *θ*_P‑Rh‑Cl_ amounts to 156.2° (red polymorph) and 166.7° (orange polymorph), and *θ*_P‑Rh‑P_ amounts to 152.8° (red) and 159.1° (orange) for the corresponding polymorphic forms.^14,15^ The distortion primarily affects the ligand-metal-ligand angles, leading to a non-planar coordination environment around the Rh center. The differences between the solid-state structures and the solution-phase optimized structure can be explained by crystal-packing effects and intramolecular interactions between bulky PPh_3_ ligands, as discussed in the literature,^15^ and may also reflect differences between the molecular models used in the theoretical calculations and the experimental solid state systems. The structural parameters optimized in vacuo differ negligibly from those obtained in solution, indicating that the nonpolar solvent exerts only minor influence on the geometric structure of this system (Table SI1). These results are also in qualitative agreement with previous theoretical studies employing the M06 ^59^ and B3LYP^60^ density functionals.

**Figure 1:**
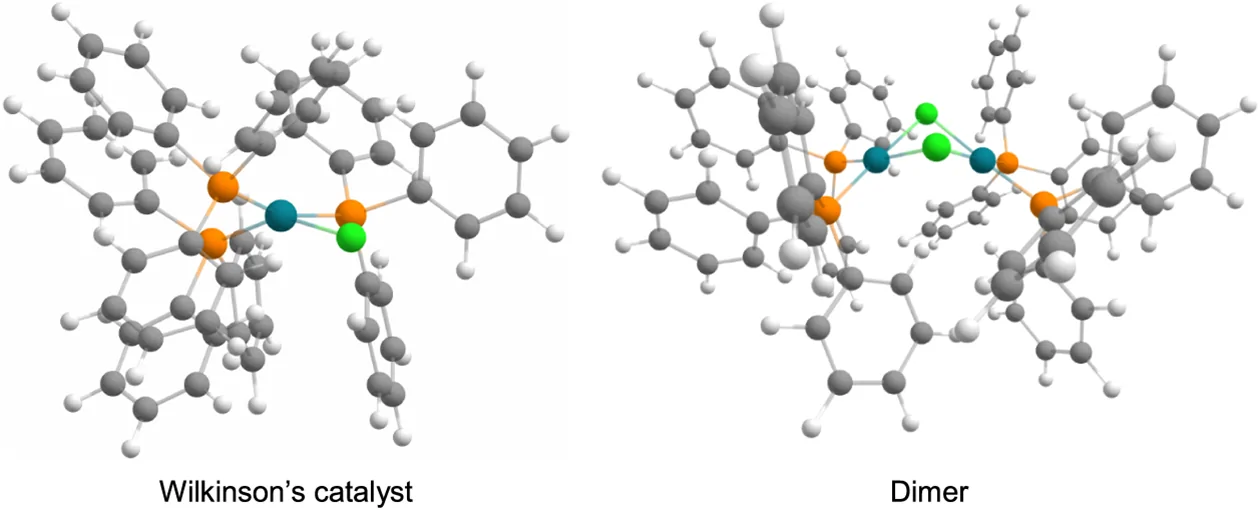
Structures of Wilkinson’s catalyst [Rh­(PPh_3_)_3_Cl] (left) and the dimer [Rh­(PPh_3_)_2_Cl]_2_ (right), in toluene, optimized at the TPSSh-D4/[def2-SVP (non-Rh) + def2-TZVPP/def2-ECP (Rh)] (COSMO, toluene) level of theory. Here and throughout, the atom colors are defined as follows: Rh —pine green, Cl—green, P—orange, C—gray, H—white.

**Figure 2:**
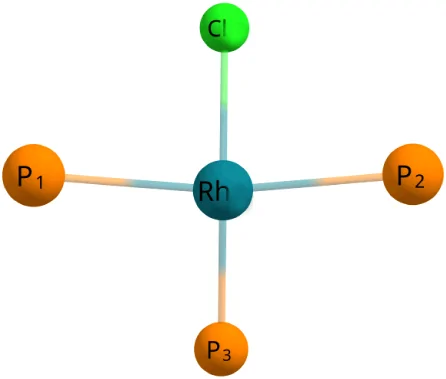
Atom numbering of the coordinating atoms in the Rh coordination environment of the complex core.

**Table 1:** Key Structural Parameters of the Investigated Complexes, [Rh­(PPh_3_)_3_Cl], [Rh­(PH_3_)_3_Cl] and [Rh­(PPh_3_)_2_Cl]_2_ at the TPSSh-D4/[def2-SVP (Non-Rh) + def2 TZVPP/def2-ECP (Rh)] (COSMO, Toluene) Level of Theory^a^

Parameter	[Rh(PPh_3_)_3_Cl]	[Rh(PPh_3_)_3_Cl] (planar)	[Rh(PH_3_)_3_Cl] (SPC)	[Rh(PPh_3_)_2_Cl]_2_
*θ*_P‑Rh‑Cl_/°	150.01 (156.2, 166.7)	176.57	179.39	171.68
*θ*_P‑Rh‑P_/°	160.94 (152.8, 159.1)	171.77	170.35	-
*φ*_Rh‑P1‑P3‑Cl_/°	21.24	0.16	0.31	0.81
*d*_P1‑Rh_/Å	2.275 (2.334. 2.304)	2.298	2.276	2.206
*d*_P2‑Rh_/Å	2.316 (2.322, 2.338)	2.334	2.282	2.223
*d*_P3‑Rh/_Å	2.230 (2.214, 2.225)	2.227	2.204	-
*d*_Cl‑Rh/_Å	2.386 (2.375, 2.404)	2.375	2.370	2.412^b^

a Atom numbering follows Figure 2. Experimental solid- state parameters are presented in parentheses for the red and orange polymorphs.

b -average

A planar conformer of Wilkinson’s catalyst monomer was also identified by semi-empirical conformer analysis (Table SI1). This form has less direct π-stacking between ligand phenyl groups (two instead of three in the distorted conformer) and is more stable by 29 kJ/mol at TPSSh-D4/[def2-SVP (non-Rh) + def2-TZVPP/def2-ECP (Rh)] (COSMO, toluene) level of theory. This planar form also exhibits a stronger asymmetric in-plane deformation, with an angle Cl–Rh–P2 angle of 83.1°. Thus, stabilization of the planar structure in the complex core appears thermodynamically favored over π-stacking stabilization. However, a larger number of diverse distorted conformers were found that are sterically hindered and stabilized by π-stacking interactions; therefore, a mixture of different kinetically stabilized forms may provide a suitable model of the real experimental system. The ordering of the conformers predicted by semiempirical methods may also differ at higher levels of theory. A detailed analysis of energetics and relationships among diverse conformers in an ensemble may provide further insight into the overall thermodynamic properties of Wilkinson’s catalyst in solution, but this lies beyond the scope of the present work.

A simplified model system, [Rh­(PH_3_)_3_Cl], in which the phenyl substituents were replaced by hydrogen atoms, adopts an almost perfectly square-planar coordination geometry around Rh with the phosphine ligands. Only minor deviations from the ideal square-planar structure are observed and can be attributed to a slight symmetry distortion of the ligand framework induced by the chlorine ligand. This structure is referred to as the square-planar complex (SPC). The most important structural parameters are summarized in Table 1 (see the atom numbering in Figure 2). The full structural data of Wilkinson catalyst (Table SI1), fragments (Table SI2), small model system in planar (Table SI3) and deformed form (Table SI4) as well as dimer structure (Table SI5) can be found in the Supporting Information.

Upon dimerization, steric effects become significantly less important, and the planar character of the coordination environment is largely restored (Figure 1). The optimized dimer structure exhibits a clear tendency toward planarity of the Rh coordination spheres, indicating that electronic effects dominate the structural preferences in the dimeric species rather than steric effects. Moreover, the dimer structure exhibits the most symmetric bonding situation between the monomer fragments, with the central structural motif close to *C*_*2v*_ point group symmetry despite the bulky PPh_3_ ligands.

The calculated harmonic vibrational spectrum of the monomer shows good agreement with experimental IR data (Figure 3 and SI1 for full spectrum) reported in the literature, particularly for modes associated with vibrations within the coordination sphere around the Rh atom. These modes correspond to the first peak in experimentally observed spectrum presented in Figure 3 and provide additional support for the electronic-structure model of Rh–P bound force constants of the complex. It is a noteworthy that, in the considered spectral range, the difference between the distorted and planar forms is negligible. This finding indicates similar metal-ligand bond strength for the identified stretching vibrations. The more intense peaks at higher wavenumbers originate from phenyl group vibrations within the ligands. The experimental ligand peaks show some shape differences due to solid-state packing effects and intermolecular interactions in the crystal as well as anharmonicity that is not accounted for in the harmonic potential approximation employed in the present calculations.

**Figure 3:**
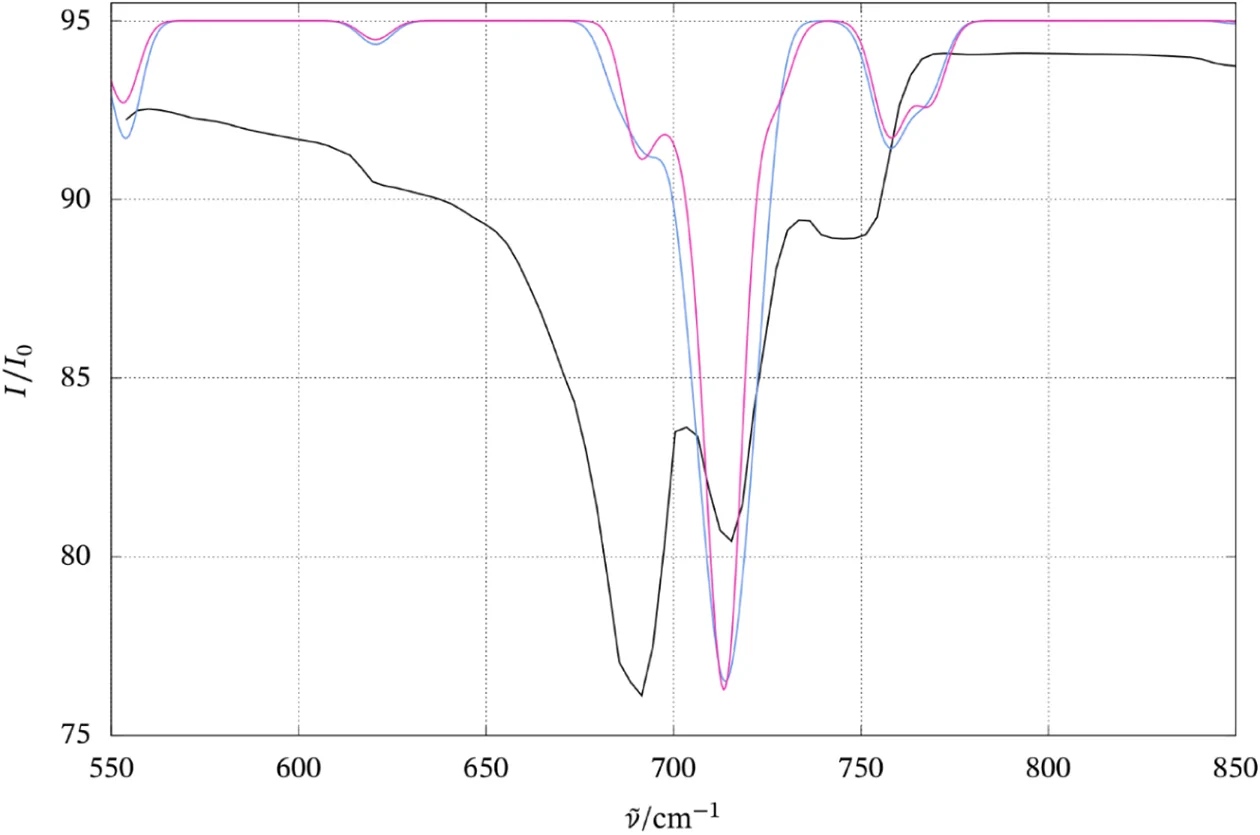
Comparison of the experimental data of solid-state IR spectrum^61^ (black) and the calculated TPSSh-D4/[def2-SVP­(non-Rh) + def2-TZVPP/def2-ECP (Rh)] (COSMO, toluene) IR spectra (blue for distorted form and magenta for the planar form) of Wilkinson’s catalyst in its monomeric form (Gaussian half-width: 4 cm^–1^).

The calculated IR spectrum of the monomer provides independent support for the proposed structure. Vibrational modes identified as associated with the coordination sphere around the Rh atom (Table 2), including metal–ligand stretching and bending motions, are reproduced with good accuracy relative to experimental data. These modes are particularly sensitive to the degree of electron delocalization and the donor–acceptor character of the Rh–PPh_3_ ligand interactions.

**Table 2:** Calculated and Experimental Values of the Most Intense IR Peaks Assigned to Vibrations of the Rh–Ligand Complex Core Values for the Planar Form are Given in Parenthesis^a^

Calculated	IR lit.^62^	IR lit.^61^
684 (689)	687	690
690 (691)		

a Values for the planar form are given in parentheses.

The agreement between calculated and experimental frequencies suggests that the applied theoretical framework captures underlying force constants associated with the proposed multicentre bonding pattern. This spectroscopic consistency further corroborates the validity of the bonding description derived from the subsequent electronic-structure analyses.

### Thermodynamic Considerations of Dimerization

The solution-phase thermodynamics of Wilkinson’s catalyst is of interest for understanding its reactivity. The dimerization reaction of Wilkinson’s catalyst in solution has been described in the literature^5^ and can be represented as two sequential reactions: dissociation of one PPh_3_ ligand from the monomer (eq 1), followed by dimerization of two [Rh­(PPh_3_)_2_Cl] fragments to form the dimer [Rh­(PPh_3_)_2_Cl]_2_ (eq 2).

1 $$\left[\mathrm{Rh}{({\mathrm{PPh}}_{3})}_{3}\mathrm{Cl}\right]\to \left[\mathrm{Rh}{({\mathrm{PPh}}_{3})}_{2}\mathrm{Cl}\right]+{\mathrm{PPh}}_{3}$$

2 $$2\left[\mathrm{Rh}{({\mathrm{PPh}}_{3})}_{2}\mathrm{Cl}\right]\to {[\mathrm{Rh}{({\mathrm{PPh}}_{3})}_{2}\mathrm{Cl}]}_{2}$$

Thermodynamic calculations demonstrate that, for the distorted conformer, the overall dimerization reaction is slightly but sufficiently, favorable in terms of Gibbs free energy (Δ*G* ^*0*^_298_ = −14 kJ mol^–1^). The favorable dimer-forming step (Δ*G* ^*0*^_298_ = −166 kJ mol^–1^) thereby more than compensates for twice the ligand dissociation free energy along the energetically preferred *cis* pathway respect to chlorine of the monomer (two times 76 kJ mol^–1^, see Table 3). For the planar form, however, the corresponding overall reaction becomes unfavorable at room temperature (Δ*G* ^*0*^_298_ = 44 kJ mol^–1^). The first ligand dissociation step from the *cis* position relative to chlorine is preferred, consistent with previous theoretical work on the distorted monomer at BP86/6-31G** level of theory with Stuttgart RECPs basis set for Rh, with *d* polarization for P and Cl.^13^

**Table 3:** Calculated Thermodynamic Parameters for the Reactions (1) and (2) at the TPSSh-D4/[def2-SVP (Non-Rh) + def2 TZVPP/def2-ECP (Rh)] (COSMO, Toluene) Level of Theory (kJ mol^–1^ and kJ (mol^–1^ K^–1^) in Toluene. Values for Planar Form are Presented in Brackets

Reaction	ΔE_el_	Δ*H* ^*0*^_298_	Δ*G* ^*0*^_298_	ΔS
(1) Wilkinson’s *trans* ligand dissociation	210 (240)	199 (228)	111 (141)	0.293 (0.292)
(1) Wilkinson’s *cis ligand dissociation*	153 (183)	147 (176)	76 (105)	0.238 (0.236)
(2) Wilkinson’s dimerization	–260	–258	–166	–0.308
(1) SPC *trans* ligand dissociation	222	215	159	0.187
(1) SPC *cis* ligand dissociation	150	142	96	0.154
(2) SPC dimerization	–234	–229	–173	–0.188

The fragment [Rh­(PPh_3_)_2_Cl] formed along the *cis* dissociation pathway is a readily identifiable monomeric building block for subsequent dimerization. It exhibits a purely electronic relaxation energy of −76 kJ mol^–1^ upon relaxation toward a planar configuration. This value is comparable with approximately half of the dissociation enthalpy along the *cis* reaction pathway and is slightly smaller than half of the corresponding pure electronic energy of this path (153 kJ mol^–1^ for full complex and 150 kJ mol^–1^ for SPC model system). Thus, up to ∼50% of the dissociation reaction energy is compensated for by the relaxation of the distorted complex core structure relaxation. Notably, the relaxation energy for the fragment formed along the trans dissociation path is larger (−90 kJ mol^–1^), which can be attributed to the lower steric hindrance of the oppositely positioned PPh_3_ ligands. Nevertheless, the *trans* dissociation path remains energetically disfavored for both considered conformers. Thus, the reaction thermodynamics for the *trans* pathway (Δ*G* ^*0*^_298_ = 56 kJ mol^–1^ for the distorted conformer) cannot be rationalized by steric effects alone but are instead dominated by bonding energetics.

These results are consistent with experimental observations of spontaneous dimerization of the complex in solutions on the assumption of kinetically stabilized distorted conformer, but they do not thermodynamically support dimerization from planar form within the considered reaction model.^5^ A consistent thermodynamic model of the dimerization for the planar conformer presumably requires an explicit description of the interaction with the solvent. The *cis* dissociation product conformer originating from planar form has weaker π-staking interactions and an enthalpy higher by 5 kJ mol^–1^. Including entropy, the corresponding free energy gain is estimated to be approximately 1 kJ mol^–1^. This conformer of *cis* dissociation pathways was therefore not considered for structural and reaction-energetic analyses.

In the simplified SPC model, [Rh­(PH_3_)_3_Cl], the metal coordination center remains essentially planar, whereas in Wilkinson’s catalyst a noticeable, sterically induced distortion of the coordination environment is present in most identified conformers. From a purely geometric relaxation standpoint, such distortion might be expected to yield a substantial energetic effect in the order of 70–90 kJ mol^–1^. However, the computed dissociation energies of Wilkinson’s catalyst and the simplified SPC model system do not differ as dramatically as this estimate would suggest.

Comparison of the reaction enthalpy points to the importance of the energy balance between metal-ligand bonding and π-stacking interactions in Wilkinson’s catalyst. The present results do not confirm earlier theoretical results of lower dissociation energies in Wilkinson’ catalyst compared with the SPC model, which were obtained from calculations without dispersion corrections; they also yield higher values than those reported in literature.^60^ This observation indicates that noncovalent interactions such as dispersion effects and weak hydrogen bonding involving the ligands, make a significant contribution to the reaction energetics. In contrast to previous discussions in literature, the π interactions between phenyl rings should therefore be regarded as stabilizing interactions despite the pronounced steric hindrance within the coordination sphere. These interactions may partially compensate for the energetic consequences of structural relaxation at the metal center. Relaxation stabilization can facilitate the formation of the monomeric fragment for kinetically stabilized distorted forms and increase the propensity for dimerization, while the non-covalent interactions have a stabilization role of Wilkinson’s catalyst itself. The energy difference between the distorted and planar isomers of Wilkinson’s catalyst supports this interpretation, indicating that the 29 kJ mol^–1^ energy gain results from favorable planarization of the complex core and unfavorable reorganization of the ligand spheres occurring simultaneously.

Steric hindrance in Wilkinson’s catalyst conformers and intermediate fragments was estimated using the percent buried volume and is presented in Table 4. The data demonstrate that the cis form of the dissociation product is less sterically hindered and therefore may be the kinetically most accessible species in catalysis from a steric point of view.

**Table 4:** Percent Buried Volume (%V_bur_) and Percent Free Volume (%V_free_) in Coordination Sphere of the Investigated Wilkinson’s-Type Systems

Molecule	%V_bur_	%V_free_
Wilkinson’s	95.4	4.6
Wilkinson’s (planar)	94.1	5.9
[Rh(PPh_3_)_2_Cl] (cis)	82.0	18.0
[Rh(PPh_3_)_2_Cl] (trans)	83.9	16.1

Comparison of the dimerization and dissociation energy balance demonstrates that the total two step dimerization process from distorted Wilkinson’s catalyst monomer (eqs 1 and 2) appears to be entropy-driven, as shown by the calculated energy balance between two dissociation reactions and dimerization step: although Δ*H* ^*0*^_298_ is positive, the Gibbs free energy Δ*G* ^*0*^_298_ is negative, indicating that the favorable entropy term dominates the thermodynamics. This effect is consistent with the formation of two fragments via the *cis* dissociation pathway, where the increase in the number of independent species provides sufficient entropic stabilization to outweigh the unfavorable enthalpic contribution.

Investigation of the coordination of two hydrogen atoms to the ligand-free catalyst fragment, a step frequently discussed in the literature^2,63^ as part of proposed hydrogenation mechanisms, suggests that trans coordination is energetically preferred (Figure 4 and SI7 in 3D). The calculated Gibbs free energy difference between the *cis*- and *trans*-coordinated hydrogen species (Table 5) is found to be larger than the corresponding free energy difference between the *cis* and *trans* dissociation pathways (Table 3).

**Figure 4:**
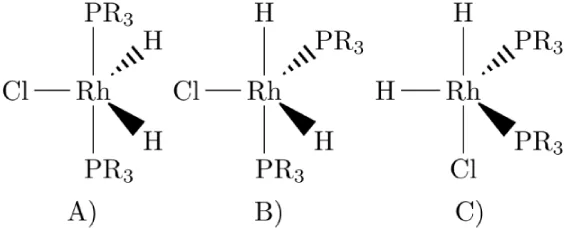
Hydrogen coordination to the Wilkinson’s catalyst fragment with one ligand removed: A) *trans;* B) *cis;* C) *syn*.

**Table 5:** Calculated Energy Differences for the Coordination of H_2_ to the Rh Center in the [Rh­(PPh_3_)_2_Cl] Fragment at TPSSh-D4/[def2-SVP (Non-Rh) + def2 TZVPP/def2-ECP (Rh)] (COSMO, Toluene) Level of Theory (kJ mol^–1^)

Coordination	Δ*H* ^*0*^_298_	Δ*G* ^*0*^_298_
*trans*	0	0
*cis*	16	35
*syn*	26	49

Within the limitations of the present computational model, this observation indicates that formation of the *trans*-coordinated intermediate may be thermodynamically favored in the catalytic cycle. However, a *cis* coordination mode followed by subsequent isomerization, analogous to pathways previously considered in the literature both experimentally and at the MP2//BP86 level of theory,^13^ cannot be ruled out.

By comparison, the *syn* coordination motif discussed in ref 2 for the second hydride-route mechanism, involving further isomerization from the *trans*-coordinated form, appears, based on our calculations, to be less thermodynamically favorable than the alternative coordination modes examined here. Accordingly, mechanisms involving the *trans*-coordination form appear preferable.

### Electronic Structure and the Nature of Chemical Bonding

Analysis of the HOMO–LUMO region in the SPC, DSPC (Figure 4), and Wilkinson’s catalyst (Figure 5) shows that deformation of the primary coordination sphere reorders the highest occupied canonical orbitals, imparting Rh–ligand bonding character to the HOMO. This electronic reorganization results in a reduction of the HOMO–LUMO gap in the distorted complex DSPC (3.06 eV) compared to the planar reference system SPC (3.77 eV). In the full structure of Wilkinson’s catalyst, the gap is further reduced (2.84 eV) due to a substantial contribution of the ligands, particularly the phenyl-containing fragments, to the character of the LUMO, which exhibits a pronounced metal-ligand antibonding nature. The reduction in the HOMO-LUMO gap between the distorted and planar isomers of Wilkinson’s catalyst is approximately 0.15 eV and is therefore much smaller than that observed for SPC compared with DSPC.

**Figure 5:**
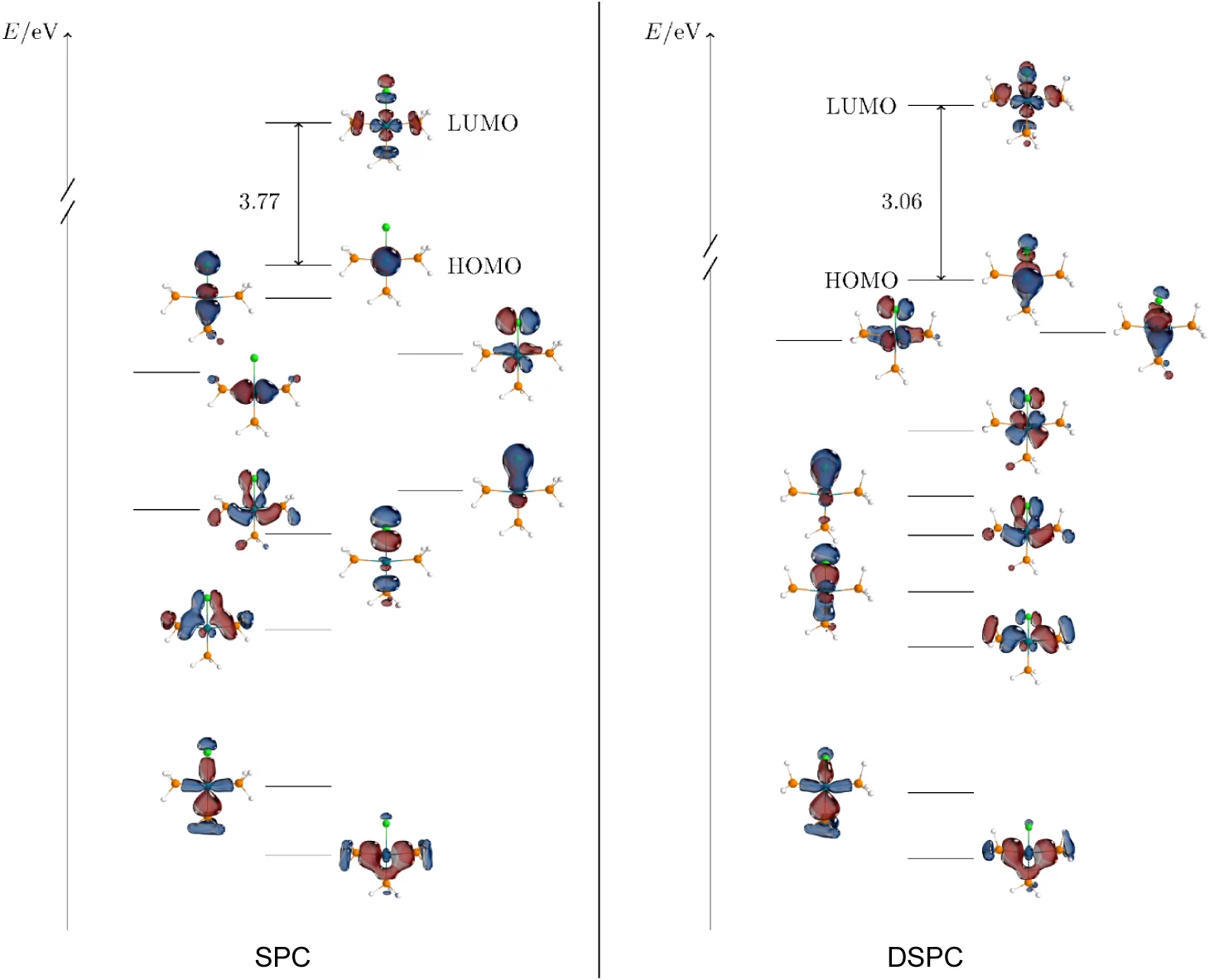
Energy diagram of the HOMO-LUMO region for the SPC and DSPC model systems at the X2C-TPSSh/[x2c-def2-TZVPall (non-Rh) + x2c-def2-TZVPPall (Rh)] level of theory.

In contrast, the electronic structure of the dimer (Figure 6) differs qualitatively from that of Wilkinson’s catalyst and the simplified model systems even at this level of analysis. The upper manifold of occupied orbitals consists predominantly of electron pairs localized on the rhodium centers, while the lowest unoccupied molecular orbital is largely ligand-centered. Notably, despite the planar configuration of the rhodium coordination spheres, the HOMO–LUMO gap in the dimer (2.67 eV) is the smallest among all systems considered. These trends are preserved across all levels of theory employed (Table SI7).

**Figure 6:**
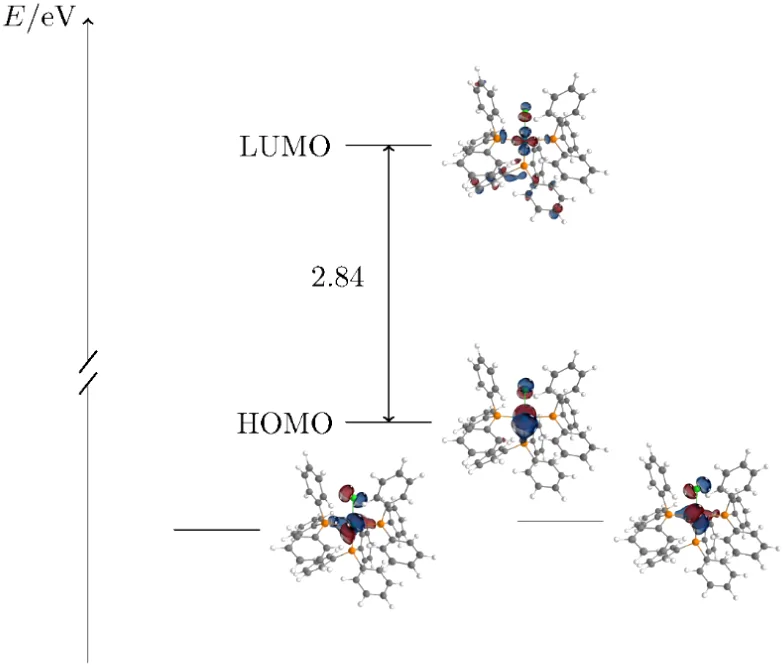
Energy diagram of the HOMO-LUMO region for distorted Wilkinson’s catalyst at the X2C-TPSSh/[x2c-def2-TZVPall (non-Rh) + x2c-def2-TZVPPall (Rh)] level of theory.

For the full monomer structure and all smaller model systems considered, the electronic structure is best described in terms of two crossed three-center, four-electron (3c–4e) bonding interactions involving the central Rh­(l) atom and pairs of ligands located on opposite sides of the coordination plane. This bonding motif cannot be adequately represented by a single classical two-center bonding scheme. These *ω* hyperbonds were consistently identified in the NBO analysis, and their composition in terms of bonding (BD) and lone-pair (LP) contributions is presented in Figures 7 and 8. This picture is consistently reproduced by all applied full electronic-structure methods, including DFT and wavefunction-based X2C-CCSD calculations for the SPC and DSPC reduced model systems. In contrast, for the dimer the NBO analysis did not identify any multicenter bonds at either the X2C-r^2^SCAN or the X2C-TPSSh level of theory.

**Figure 7:**
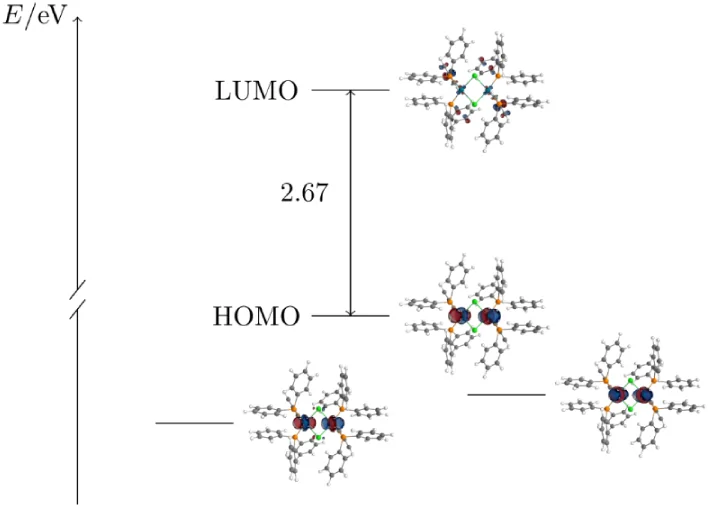
Energy diagram of the HOMO-LUMO region for the dimer at the X2C-TPSSh/[x2c-def2-TZVPall (non-Rh) + x2c-def2-TZVPPall (Rh)] level of theory.

**Figure 8:**
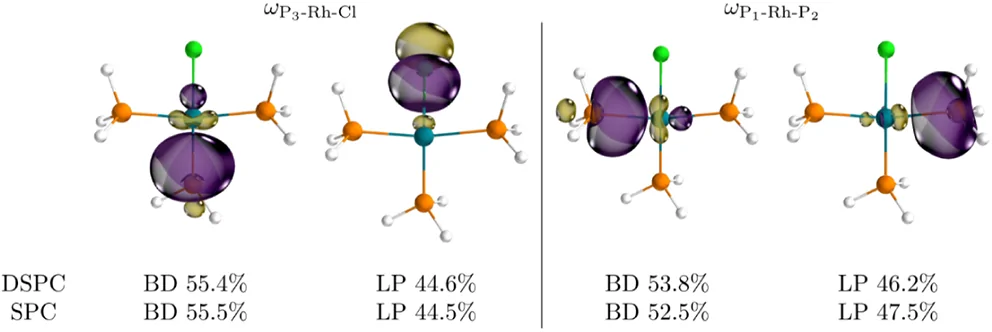
Structures and compositions of the 3c–4e *ω* hyperbonds in the SPC and DSPC model systems at the X2C-TPSSh/[x2c-def2-TZVPall (non-Rh) + x2c-def2-TZVPPall (Rh)] level of theory, namely ω­(P_3_–Rh–Cl) and ω­(P_1_–Rh–P_2_). The percentage contributions of the bonding (BD) and lone-pair (LP) components are given for each hyperbond. The DSPC structure is shown.

In addition to standard NBO analysis, Natural Resonance Theory (NRT) reveals significant contributions from multiple resonance structures corresponding to these two crossed 3c–4e bonding patterns (Figure 9 and Table 6). For both intersecting ω-bonds, approximately equal contributions from two resonance structures that differ in the localization of opposite electron pairs are observed in the description of the bonding electronic structure. All four resonance structures collectively account for the bonding pattern within the core of the complex, which can be described as two simultaneously present and mutually intersecting three-center four-electron (3c–4e) bonds centered on the metal atom. Collectively, this bonding pattern involves eight electrons delocalized over five centers, along with eight electrons identified as nonbonding lone pairs localized on the rhodium atom. Taken together, these features are fully consistent with the well-established 16-electron rule for complexes of this type. The crossed three-center four-electron bonding pattern observed in the monomeric complex represents a fundamentally non-classical bonding situation that cannot be reduced to a superposition of localized two-center interactions. In this description, the Rh atom simultaneously participates in two orthogonal donor–acceptor interactions involving ligand pairs arranged trans to each other, resulting in a delocalized electronic structure stabilized by symmetry and orbital complementarity. The electronic structure of the planar isomer of Wilkinson’s catalyst is similar to that of the other monomeric systems discussed above, and a detailed comparison of numerical descriptors, such as NPA charges, reveals trends similar to those observed for the DSPC and SPC model systems.

**Figure 9:**
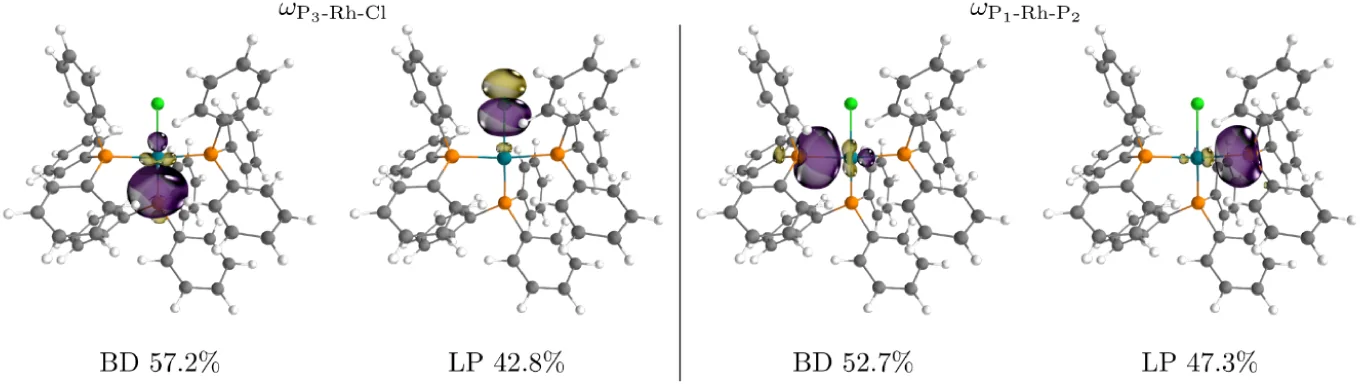
Structure of 3c–4e *ω* hyperbonds in Wilkinson’s catalyst at the X2C-TPSSh/[x2c-def2-TZVPall (non-Rh) + x2c-def2-TZVPPall (Rh)] level of theory, namely ω­(P_3_–Rh–Cl) and ω­(P_1_–Rh–P_2_). The percentage contributions from bonding (BD) and lone-pair (LP) components are given for each hyperbond.

**Table 6:** Calculated Weights (%) of the Monomeric Resonance Structures (A–D) in Figure 9 and the Dimeric Resonance Structures (A–C) in Figure 10 at the X2C-TPSSh/[x2c-def2 TZVPall (Non-Rh) + x2c-def2 TZVPPall (Rh)] Level of Theory

Resonance	SPC	DSPC	Wilkinson’s catalyst	Wilkinson’s catalyst planar	Dimer
A)	25.7	28.9	28.1	27.1	34.7
B)	25.5	22.3	24.9	24.5	18.4
C)	18.8	20.8	13.4	13.8	13.2
D)	19.3	18.1	19.0	19.5	-

This three-center bonding pattern also remains in the [Rh­(PPh_3_)_2_Cl] fragments after dissociation along the *cis* and *trans* pathways. The thermodynamically preferred product has a higher total bond order between the Rh atom and the P and Cl ligands (MBO 3.16 for *cis* vs 2.62 for *trans*) that demonstrates thermodynamic control consistent with the bonding characteristics of the complex core, the full data of bonding analysis including additionally WBI and NLMO/NPA is provided in the Supporting Information for TPSSh (Tables SI17) and r^2^SCAN (Tables SI18) functionals. The HOMO-LUMO gaps of these isomers also differ: 2.52 eV for *cis* and 2.74 eV for *trans,* indicating lower electron-density mobility in the *trans* form due to absence of back-donation opposite to chlorine and the approximately twofold higher ionic character of the Rh–Cl bond according to NRT analysis. Both fragments were characterized by resonance structures including additional lone pair localization at the Rh atom, corresponding to a localized 4d^10^ electronic structure. The resonance structures of separate *cis* (Figures SI5) and *trans* (Figures SI6) fragments and the weights of the structures (Table SI22) are provided additionally in Supporting Information Figure 10.

**Figure 10:**
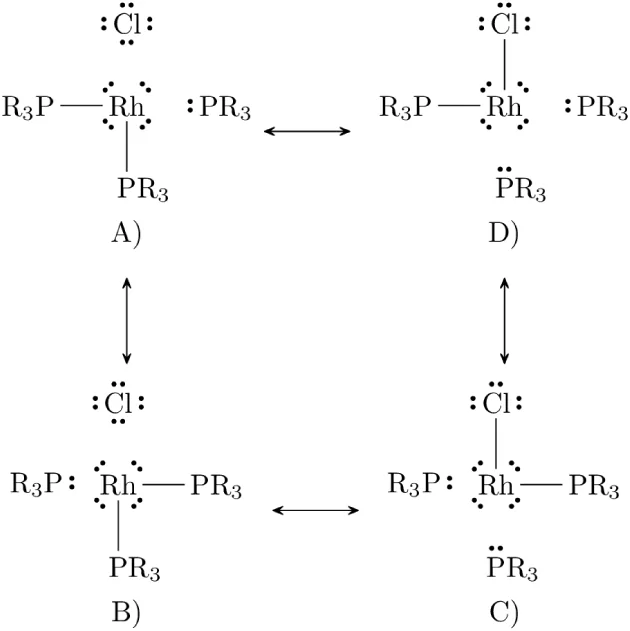
Identified natural resonance structures (A–D) of the Rh complex core in the monomeric species.

In the fragment formed along the *trans* dissociation pathway, a significant interaction between one phenyl group of the phosphine ligand and the rhodium center is observed, occupying the vacant site trans to chloride. The Rh–H distance of 1.749 Å and the adjacent Rh–C contact at 2.231 Å are within ranges commonly associated with agostic interactions in organometallic rhodium complexes (short metal-H distance in agostic species typically fall within the range of 1.8–2.3 Å) and with metal–ligand bond lengths observed in structurally characterized rhodium hydrides such as [HRh­(CO)­(PPh_3_)_3_] (Rh–H = 1.60 Å) and related systems.^64,65^ A quantitative bonding analysis likewise indicates stabilizing Rh–(C–H/aryl) bonding contributions in this fragment; nevertheless, this stabilization is insufficient to compensate for the energetic advantage associated with the three-center interactions in the P–Rh–Cl motif retained in the fragment formed upon dissociation from the *cis* configuration. The study of the resonance structure of this fragment demonstrates a weak multicenter interaction, with the dominated resonance structure featuring a lone pair on the Rh atom and a C–H bond (Figure 11). The relation between the A, B, and C resonance structures are 72.29/15.33/12.38 at the all electron TPSSh level of theory. The NBOs showing weak localization between the CH fragment and Rh are demonstrated in Figure 12.

**Figure 11:**
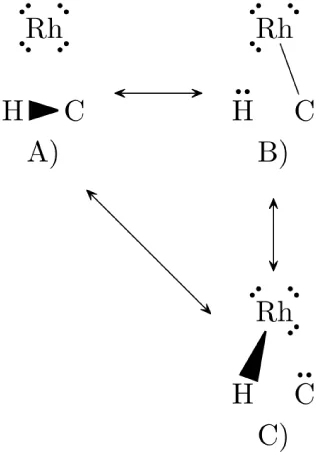
Identified natural resonance structures (A–C) of the [Rh­(PPh_3_)_2_Cl] fragment involved in the agostic interaction along the *trans* dissociation pathway.

**Figure 12:**
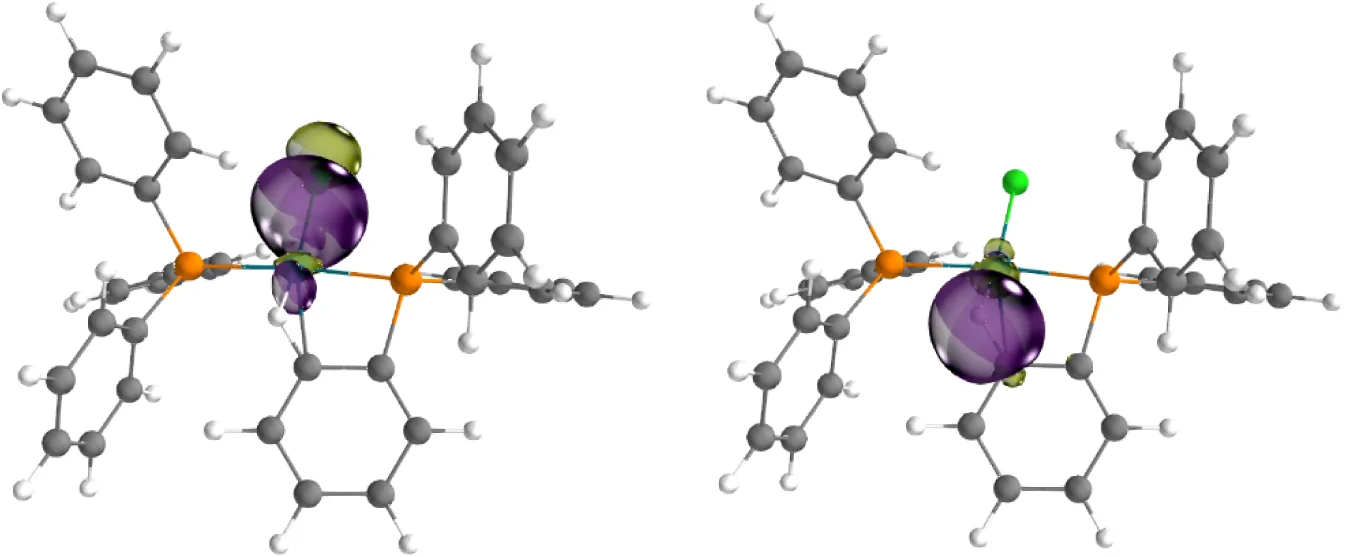
Identified bonding NBOs in the [Rh­(PPh_3_)_2_Cl] fragment associated with the agostic interaction along the *trans* dissociation pathway.

In contrast, no analogous crossed multicenter bonding pattern is found for the dimeric species. Instead, the electronic structure of the dimer is dominated by a single prevailing resonance structure, indicating a qualitative change in the nature of chemical bonding upon dimerization (Figure 13 and Table 6). Modeling of the resonance structures for all eight involved centers confirms the predomination of the localized bonded dimer, while also demonstrating a highly symmetric bonding character between the chlorine and Rh atoms, including donation and back-donation between two Rh centers, which reduces the ionic charges localized on the individual atoms. The loss of the 3c–4e bonding character in the dimer may explain the enthalpically unfavorable character of this reaction, which originates from the nature of the chemical bonding character at the electronic level.

**Figure 13:**
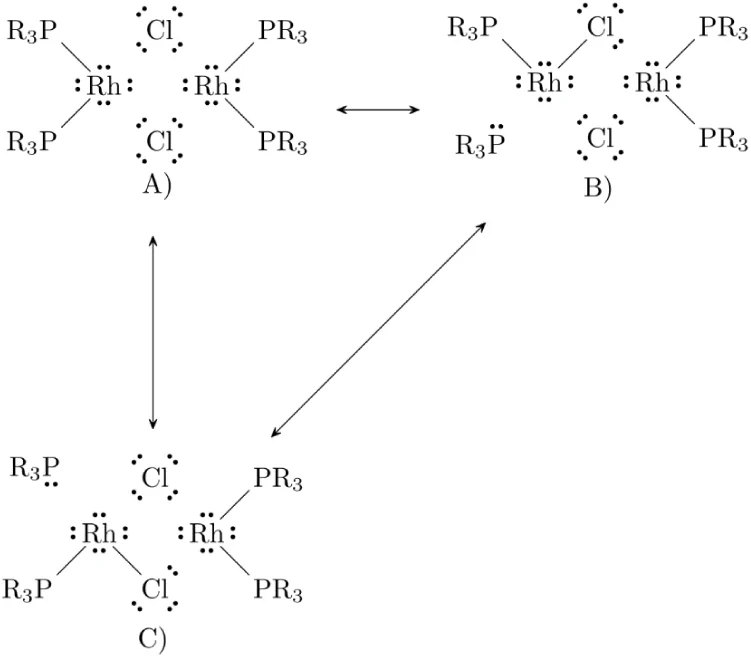
Identified natural resonance structures (A–C) of the Rh complex core in the dimeric species (simplified one-center model).

The presence of bonding interactions between the Rh atom and all four ligands is further supported by QTAIM analysis (Figures 14 and 15), which identifies bond critical points (BCPs) for each Rh–ligand interaction in all considered structures, including the dimer. The associated topological parameters indicate donor–acceptor interactions with a substantial degree of electron delocalization between atomic centers. In the dimer, a ring critical point (RCP) is found in the cavity between the Rh atoms and Cl atoms; importantly, no Rh–Rh BCP or bond path is detected, which argues against the presence of a Rh–Rh intermetallic bonding interaction within this four-atom motif. An additional noteworthy observation is the presence of electron-density BCPs between the central Rh atom and the phenyl fragments in Wilkinson’s catalyst outside the main coordination planes, as well as recognizable π-stacking between the phenyl groups of the *cis* and *trans* ligands. These weak bonding interactions suggest a possible additional stabilizing role of the ligands’ hydrogen atoms, arising from direct interactions with the nonbonding electron pairs of the distorted complex core. The coordination of Rh to the *ortho* hydrogen atoms of the phenyl group has also been discussed in the literature based on X-ray structural data for the red and orange polymorphic forms.^15^ This hydrogen-assisted coordination may also be important for molecular structural parameters, such as ligand angles in different allotropic forms and in the isolated molecule.

**Figure 14:**
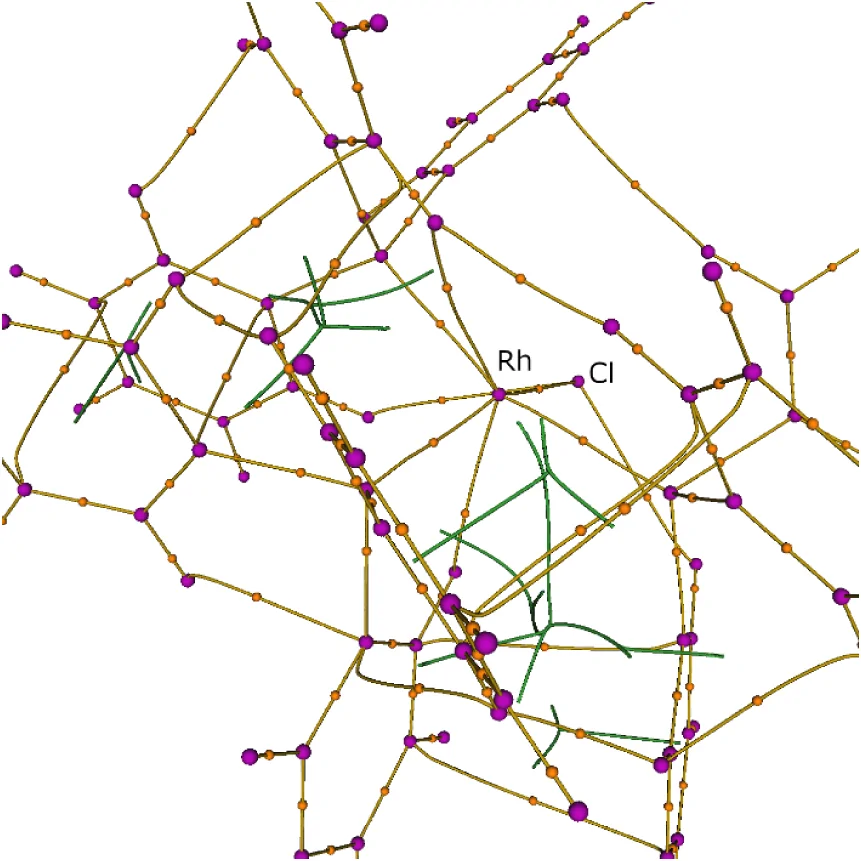
QTAIM bond topology around the core of Wilkinson’s catalyst at the X2C-TPSSh/[x2c-def2-TZVPall (non-Rh) + x2c-def2-TZVPPall (Rh)] level of theory. Violet spheres denote nuclear critical points; orange spheres denote bond critical points (BCPs). Ring critical points (RCPs) of the phenyl rings are omitted for clarity.

**Figure 15:**
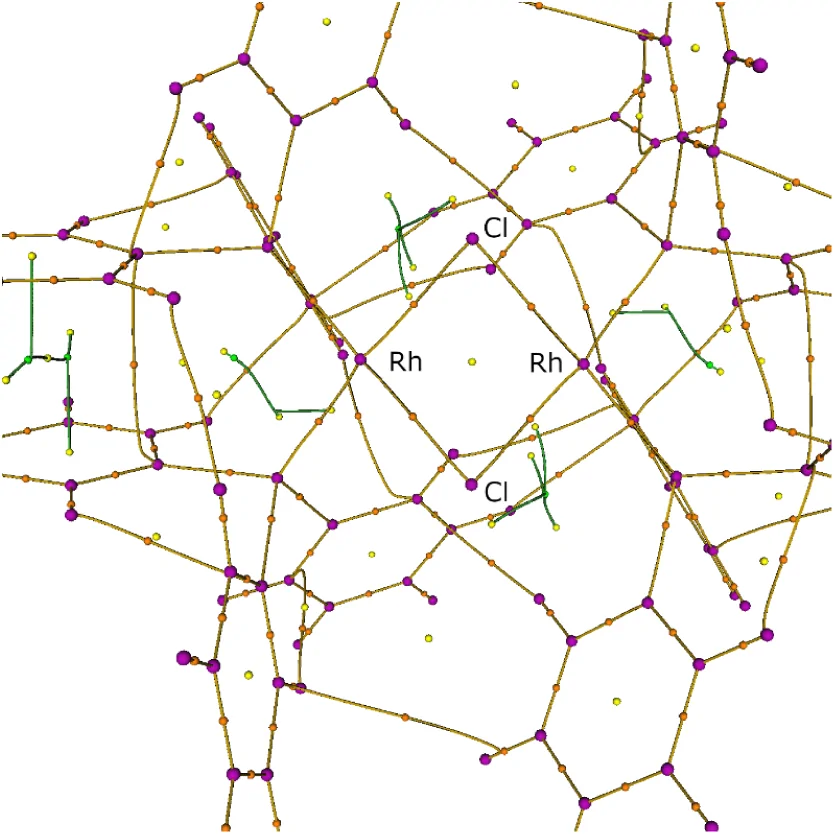
QTAIM bond topology around the core of the dimer at the X2C-TPSSh/[x2c-def2-TZVPall (non-Rh) + x2c-def2-TZVPPall (Rh)] level of theory (top view). Yellow spheres denote ring critical points (RCPs).

The π-stacking pattern was analyzed using non-covalent interaction analysis based on the reduced density gradient (RDG), combined with mapping of the density Hessian eigenvalues presented in the (Supporting Information Figure SI8). The data were produced using the Multiwfn software. The resulting visual pattern shows clearly recognizable RDG features between phenyl rings in both considered conformers of Wilkinson’s catalyst: three in the distorted conformer and two in the planar conformer. The double π-staking between ligands in *cis* dissociated fragment is also clearly recognizable, whereas no stacking is present for the more distant PPh_3_ ligands in the *trans* fragment. Moreover, the RDF/sing­(λ_2_ρ) plot demonstrated areas of high attraction around Rh atom for both conformers associated with direct interaction of Rh with phenyl groups involving hydrogens also indicated in QTAIM.

Additional analysis of NPA charges (Table 7), resonance structures, and bond orders confirms that Rh­(I) acts as a Lewis acid in all investigated systems (i.e., as an electron acceptor in donor-acceptor Rh–ligand interactions) with only minor delocalization of negative charge between Rh and chlorine. The charge distribution and orbital populations are consistent with a d^8^ Rh center and the conventional 16-electron rule for square-planar complexes of this type. This electronic configuration is preserved in the dimer; however, the Rh interaction with each individual chlorine ligand is weakened due to increased delocalization of chlorine electron pairs between the two Rh centers. As a consequence, the NPA charge on chlorine becomes smaller. The Rh–Cl-Rh bond angle indicates that orbital orientation and electron-pair repulsion play a dominant role in determining the dimer geometry. All P atoms of all ligands carry positive NPA charges.

**Table 7:** Calculated NPA Charges for the Monomeric Model Systems Calculated at the X2C-TPSSh/[x2c-def2 TZVPall (Non-Rh) + x2c-def2 TZVPPall (Rh)] Level of Theory

	SPC	DSPC	Wilkinson‘s catalyst
Rh	–0.43	–0.39	–0.38
P2	0.22	0.26	1.10
P1	0.30	0.30	1.14
P3	0.37	0.29	1.13
Cl	–0.54	–0.56	–0.56

Quantitative analysis of chemical bonding based on Wiberg bond indices (WBI), Mayer bond orders (MBO), and Natural Localized Molecular Orbitals (NLMO)/Natural Population Analysis (NPA) was performed (Tables 8 and 9). These descriptors indicate that MBO provide a representation of bonding in the complex that is closest to chemical intuition; however, they are less sensitive to differences between the complexes. In contrast, WBI tend to be smaller than would be expected from classical bond-order concepts without additional normalization. From this analysis, it is evident that the Rh–P bond trans to the Cl atom is stronger than the Rh–P bond involving the *cis*-coordinated PR_3_ ligand, which indirectly confirms the higher dissociation energy from the *trans* position. This difference is most apparent in the WBI values, however, all approaches considered show the same trend. In addition, the Rh–P bond order in the dimer is much higher than in all distorted monomers. Calculation of the force constants for the considered Rh–P bonds exhibits a direct correlation between the presented bond order/bond index and the bond strength calculated at the TPSSh-D4/[def2-SVP (non-Rh) + def2-TZVPP/def2-ECP (Rh)] level of theory and presented in additional table (Table SI16).

**Table 8:** Calculated Quantitative Bond Indices for the Monomeric Model Systems Calculated at the X2C-TPSSh/[x2c-def2 TZVPall (Non-Rh) + x2c-def2 TZVPPall (Rh)] Level of Theory

	Rh–P1	Rh–P2	Rh–P3	Rh–Cl
SPC				
MBO	0.83	0.82	1.09	0.74
WBI	0.52	0.52	0.65	0.40
NLMO	0.52	0.34	0.60	0.30

	Rh–P1	Rh–P2	Rh–P3	Rh–Cl
DSPC				
MBO	0.88	0.81	1.08	0.80
WBI	0.55	0.50	0.65	0.41
NLMO	0.54	0.33	0.57	0.29

	Rh–P1	Rh–P2	Rh–P3	Rh–Cl
Wilkinson’s catalyst				
MBO	0.83	0.96	0.99	0.90
WBI	0.51	0.46	0.61	0.36
NLMO	0.55	0.36	0.60	0.25

**Table 9:** Calculated Quantitative Bond Indices for the Dimer Calculated at the X2C-TPSSh/[x2c-def2 TZVPall (Non-Rh) + x2c-def2 TZVPPall (Rh)] Level of Theory (the Indices for the Second Rh Atom are Symmetry-Equivalent)

	Rh1–P1	Rh1–P2	Rh1–Cl1	Rh1–Cl2
MBO	1.05	1.15	0.47	0.45
WBI	0.67	0.65	0.30	0.32
NLMO	0.65	0.63	0.21	0.22

Additional insight into the qualitative difference between the monomeric and dimeric species is provided by the analysis of NLMOs. For the monomeric complex, the presence of crossed three-center, four-electron interactions leads to pronounced difficulties in orbital localization for each of the 3c–4e *ω* bonds. The resulting NLMOs exhibit distorted localization symmetry and symmetry breaking in the NLMO bonding pattern, reflecting the intrinsically delocalized nature of the bonding framework and the inadequacy of a strictly localized two-center description. A similar electronic structure with localized metal-ligand bonds has been described in the literature for chlorine-bridged Zr complex dimers.^66,67^

In sharp contrast, the dimeric species displays a largely classical and localized bonding pattern. In this case, NLMO localization proceeds unambiguously and yields well-defined orbitals consistent with conventional two-center bonding descriptors. The symmetry properties of the NLMOs in the dimer further support this interpretation, as they closely follow the molecular symmetry and do not exhibit the localization instabilities observed for the monomer.

These observations provide an orbital-based illustration of the qualitative change in chemical bonding upon dimerization. While the monomer is characterized by inherently delocalized multicenter interactions that resist localization, the dimer adopts a bonding regime that is more electronically localized and readily interpretable within classical bonding concepts.

The NBO analysis of the bonding pattern in systems containing coordinated hydrogen, [H_2_Rh­(PPh_3_)_2_Cl], did not reveal evidence for multicenter bonding along the metal-hydrogen coordination directions. In contrast, three-center four-electron (3c–4e) bonding interactions remain identifiable between ligands mutually arranged in *trans* positions. Comparison of the bonding patterns in the optimized structures suggests that the preference for hydrogen coordination in the *trans* position is governed primarily by steric factors, because the total bond order in this fragment is lower than that for *cis*-coordinated species, making a simple bond-strength argument inapplicable in this case that is recognizable in visualized NBOs (Figure SI9). In addition to steric factors, the total bond order in the *syn* form is slightly higher for all bond indices except WBI for TPSSh (Tables SI19) as well as for r^2^SCAN (Table SI20), however, the absence of multicenter bonding between the ligands in this form makes this coordination mode less energetically favorable than the *trans* arrangement. This finding supports the importance of the 3c–4e bonding for the thermodynamic stability of the coordinated systems and is consistent with the corresponding observations for the dimerization enthalpy.

## Conclusion

A combined DFT and wavefunction-based study of Wilkinson’s catalyst has been presented. Steric effects, non-covalent interactions, and π-stacking between phenyl groups of the bulky ligands are shown to control deviations from an ideal square-planar geometry and the energetics of the π-stacking controlled monomeric form, while purely electronic factors dominate the geometric structure of the dimeric species and the enthalpy balance of all reaction steps. A thermodynamically stable planar conformer of Wilkinson’s catalyst has been demonstrated and identified as a form without a thermodynamic preference for dimerization. Bonding analyses reveal that the monomer is best described by two crossed three-center, four-electron (3c–4e) interactions, a feature absent in the dimer, indicating a qualitative change in bonding upon dimerization. The central Rh atom consistently functions as an electron acceptor (i.e., as a Lewis acid) in donor-acceptor Rh–ligand interactions and remains consistent with the 16-electron electron count rule in both monomeric and dimeric forms. The thermodynamics of Wilkinson’s catalyst is in agreement with the bonding character of the dissociation pathways and is controlled by electronic structure factors associated with chemical bonding, while the dimerization appears to be purely entropy-controlled and preferred at room temperature, supporting the experimentally observed tendency toward dimerization in solution. The coordination thermodynamics of hydrogen in the investigated catalytic intermediates appears to be governed primarily by steric effects, in contrast to the inter- and intramolecular reactions involving Wilkinson’s catalyst. These findings highlight the importance of multicenter bonding concepts for understanding the structure and reactivity of Wilkinson’s catalyst.

## Supplementary Material



## Notes

Structural coordinates and computational data for Wilkinson’s catalyst; model fragments and dimer; IR spectra; energy HOMO-LUMO data; resonance and bonding analyses; QTAIM and NPA results; bond orders; RDG plots; and NBO visualizations (PDF)

## References

[1] Medici S., Peana M., Pelucelli A., Zoroddu M. A. (2021). Rh­(I) Complexes
in Catalysis: A Five-Year Trend. Molecules.

[2] Nelson D. J., Li R., Brammer C. (2005). Using Correlations
to Compare Additions to Alkenes:
Homogeneous Hydrogenation by Using Wilkinson’s Catalyst. J. Org. Chem..

[3] Wilkinson’s Catalyst Comprehensive Organic Name Reactions And Reagents; Wiley, 2010 pp. 3011–3017. DOI: 10.1002/9780470638859.conrr671

[4] Young J. F., Osborn J. A., Jardine F. H., Wilkinson G. (1965). Hydride Intermediates
in Homogeneous Hydrogenation Reactions of Olefins and Acetylenes Using
Rhodium Catalysts. Chem. Commun..

[5] Osborn J. A., Jardine F. H., Young J. F., Wilkinson G. (1966). The Preparation
and Properties of Tris­(Triphenylphosphine)­Halogenorhodium­(I) and Some
Reactions Thereof Including Catalytic Homogeneous Hydrogenation of
Olefins and Acetylenes and Their Derivatives. J. Chem. Soc. A.

[6] Evans D., Osborn J. A., Wilkinson G. (1968). Hydroformylation
of Alkenes by Use
of Rhodium Complex Catalysts. J. Chem. Soc.
A.

[7] Männig D., Nöth H. (1985). Catalytic
Hydroboration with Rhodium Complexes. Angew.
Chem., Int. Ed..

[8] Onopchenko A., Sabourin E. T., Beach D. L. (2002). Rhodium­(I)-Catalyzed Hydrosilylation
of Styrene. J. Org. Chem..

[9] Ohno K., Tsuji J. (2002). Organic Synthesis by Means of Noble
Metal Compounds. XXXV. Novel
Decarbonylation Reactions of Aldehydes and Acyl Halides Using Rhodium
Complexes. J. Am. Chem. Soc..

[10] Edel K., Yang X., Ishibashi J. S. A., Lamm A. N., Maichle-Mössmer C., Giustra Z. X., Liu S. -Y., Bettinger H. F. (2018). The Dewar
Isomer of 1,2-Dihydro-1,2-azaborinines: Isolation, Fragmentation,
and Energy Storage. Angew. Chem. Int. Ed..

[11] Dachs A., Torrent A., Pla-Quintana A., Roglans A., Jutand A. (2009). Rates and
Mechanism of Rhodium-Catalyzed [2+2+2] Cycloaddition of Bisalkynes
and a Monoalkyne. Organometallics.

[12] Wender P. A., Husfeld C. O., Langkopf E., Love J. A. (1998). First Studies of
the Transition Metal-Catalyzed [5+2] Cycloadditions of Alkenes and
Vinylcyclopropanes: Scope and Stereochemistry. J. Am. Chem. Soc..

[13] Goodman J., Grushin V. V., Larichev R. B., Macgregor S. A., Marshall W. J., Roe D. C. (2010). Fluxionality of [(Ph_3_ P)_3_ M­(X)] (M = Rh, Ir). The Red and Orange Forms of [(Ph_3_ P)_3_ Ir­(Cl)]. Which Phosphine Dissociates Faster
from Wilkinson’s Catalyst?. J. Am. Chem.
Soc..

[14] Choinopoulos I., Papageorgiou I., Coco S., Simandiras E., Koinis S. (2012). Modification of Wilkinson’s
Catalyst with Triphenyl
Phosphite: Synthesis, Structure, 31P NMR and DFT Study of Trans-[RhCl­(P­(OPh)­3)­(PPh3)­2]. Polyhedron.

[15] Bennett M. J., Donaldson P. B. (2002). Crystal and Molecular Structure of
the Orange and Red
Allotropes of Chlorotris­(Triphenylphosphine)­Rhodium­(I). Inorg. Chem..

[16] Jitonnom W., Wanjai T., Oláh J., Linnolahti M., Jitonnom J. (2025). Steric and Electronic Effects of Zirconocenealkyl-Borate
Ion Pairs on Catalyst Activation: A Theoretical Study. ACS Omega.

[17] Meelua W., Wanjai T., Jitonnom J. (2025). Exploring the Effect
of Zr/B Ratio
on the Stability and Reactivity of Activated ε-Caprolactone
Complexes: A DFT, QTAIM and NCI Study. J. Mol.
Graphics Model..

[18] Jitonnom W., Wanjai T., Linnolahti M., Jitonnom J. (2025). Origin of Comonomer
Reactivity in Zirconocene-Mediated Polymerization of 2-Oxazoline and
ε-Caprolactone: Insights from DFT. Organometallics.

[19] Franzke Y. J., Holzer C., Andersen J. H., Begušić T., Bruder F., Coriani S., Sala F. D., Fabiano E., Fedotov D. A., Fürst S. (2023). TURBOMOLE: Today and
Tomorrow. J. Chem. Theory Comput..

[20] Ahlrichs, R. ; Armbruster, M. K. ; Bachorz, R. A. ; Bahmann, H. ; Baldes, A. ; Bär, M. ; Baron, H. ; Bauernschmitt, R. ; Bischoff, F. A. ; Böcker, S. Turbomole; https://www.turbomole.org. accessed 08 DEcember 2026

[21] Weigend F. (2002). A Fully Direct
RI-HF Algorithm: Implementation, Optimised Auxiliary Basis Sets, Demonstration
of Accuracy and Efficiency. Phys. Chem. Chem.
Phys..

[22] Tao J., Perdew J. P., Staroverov V. N., Scuseria G. E. (2003). Climbing the Density
Functional Ladder: Nonempirical Meta–Generalized Gradient Approximation
Designed for Molecules and Solids. Phys. Rev.
Lett..

[23] Staroverov V. N., Scuseria G. E., Tao J., Perdew J. P. (2004). Erratum: “Comparative
Assessment of a New Nonempirical Density Functional: Molecules and
Hydrogen-Bonded Complexes” [J. Chem. Phys. 119, 12129 (2003)]. J. Chem. Phys..

[24] Caldeweyher E., Mewes J. -M., Ehlert S., Grimme S. (2020). Extension and Evaluation
of the D4 London-Dispersion Model for Periodic Systems. Phys. Chem. Chem. Phys..

[25] Pracht P., Grimme S., Bannwarth C., Bohle F., Ehlert S., Feldmann G., Gorges J., Müller M., Neudecker T., Plett C. (2024). CREST—A Program
for the Exploration of Low-Energy Molecular Chemical Space. J. Chem. Phys..

[26] Schäfer A., Horn H., Ahlrichs R. (1992). Fully Optimized Contracted Gaussian
Basis Sets for Atoms Li to Kr. J. Chem. Phys..

[27] Weigend F., Ahlrichs R. (2005). Balanced Basis Sets
of Split Valence, Triple Zeta Valence
and Quadruple Zeta Valence Quality for H to Rn: Design and Assessment
of Accuracy. Phys. Chem. Chem. Phys..

[28] Jitonnom W., Wanjai T., Linnolahti M., Jitonnom J. (2025). Ligand Structure Effects
on the Stability of Cationic Zirconocene Dimethylaniline Complexes:
Insights from Density Functional Theory. ACS
Omega.

[29] Klamt A., Schüürmann G. (1993). COSMO: A New Approach to Dielectric
Screening in Solvents with Explicit Expressions for the Screening
Energy and Its Gradient. J. Chem. Soc., Perkin
Trans. 2.

[30] Lunkenheimer B., Köhn A. (2013). Solvent Effects
on Electronically Excited States Using
the Conductor-Like Screening Model and the Second-Order Correlated
Method ADC(2). J. Chem. Theory Comput..

[31] Khani S. K., Khah A. M., Hättig C. (2018). COSMO-RI-ADC­(2)
Excitation Energies
and Excited State Gradients. Phys. Chem. Chem.
Phys..

[32] Peng D., Reiher M. (2012). Exact Decoupling of
the Relativistic Fock Operator. Theor. Chem.
Acc..

[33] Pollak P., Weigend F. (2017). Segmented Contracted
Error-Consistent Basis Sets of
Double- and Triple-ζ Valence Quality for One- and Two-Component
Relativistic All-Electron Calculations. J. Chem.
Theory Comput..

[34] Furness J. W., Kaplan A. D., Ning J., Perdew J. P., Sun J. (2020). Accurate and
Numerically Efficient r^2^ SCAN Meta-Generalized Gradient
Approximation. J. Phys. Chem. Lett..

[35] Scuseria G. E., Scheiner A. C., Lee T. J., Rice J. E., Schaefer H. F. (1987). The Closed-Shell
Coupled Cluster Single and Double
Excitation (CCSD) Model for the Description of Electron Correlation.
A Comparison with Configuration Interaction (CISD) Results. J. Chem. Phys..

[36] Stanton, J. F. ; Gauss, J. ; Cheng, L. ; Harding, M. E. ; Matthews, D. A. ; Szalay, P. G. ; Auer, A. A. ; Bartlett, R. J. ; Benedikt, U. ; Berger, C. ; . CFOUR, a Quantum Chemical Program Package; http://www.cfour.de.

[37] Matthews D. A., Cheng L., Harding M. E., Lipparini F., Stopkowicz S., Jagau T. -C., Szalay P. G., Gauss J., Stanton J. F. (2020). Coupled-Cluster Techniques for Computational
Chemistry:
The CFOUR Program Package. J. Chem. Phys..

[38] Harding M. E., Metzroth T., Gauss J., Auer A. A. (2008). Parallel Calculation
of CCSD and CCSD­(T) Analytic First and Second Derivatives. J. Chem. Theory Comput..

[39] Helgaker, T. ; Aa, H. J. ; Jensen, P. J. ; Olsen, J. https://cfour.uni-mainz.de. (accessed 08 December 2026).

[40] Almlöf, J. ; Taylor, P. R. https://cfour.uni-mainz.de (accessed 08 December 2026).

[41] Almlöf, J. The MOLECULE Integral Program, Technical Report No. 74-09, Institute Of Physics; University Of Stockholm, Stockholm. 1974

[42] Taylor, P. R. https://cfour.uni-mainz.de (accessed 08 December 2026).

[43] Mitin, A. V. ; Wüllen, C. V. https://cfour.uni-mainz.de (accessed 08 December 2026).

[44] Zou, W. https://github.com/zorkzou/Molden2AIM (accessed 08 December 2026).

[45] Bader R. F. W. (1991). A Quantum
Theory of Molecular Structure and Its Applications. Chem. Rev..

[46] Weinhold F. (2012). Natural Bond
Critical Point Analysis: Quantitative Relationships between Natural
Bond Orbital-based and QTAIM-based Topological Descriptors of Chemical
Bonding. J. Comput. Chem..

[47] Lu T. (2024). A Comprehensive
Electron Wavefunction Analysis Toolbox for Chemists, Multiwfn. J. Chem. Phys..

[48] Lu T., Chen F. (2012). Multiwfn: A Multifunctional
Wavefunction Analyzer. J. Comput. Chem..

[49] Falivene L., Cao Z., Petta A., Serra L., Poater A., Oliva R., Scarano V., Cavallo L. (2019). Towards the Online Computer-Aided
Design of Catalytic Pockets. Nat. Chem..

[50] Glendening, E. D. ; Badenhoop, J. K. ; Reed, A. E. ; Carpenter, J. E. ; Bohmann, J. A. ; Morales, C. M. ; Karafiloglou, P. ; Landis, C. R. ; Weinhold, F. . NBO; https://nbo6.chem.wisc.edu

[51] Glendening E. D., Landis C. R., Weinhold F. (2019). NBO 7.0: New
Vistas in Localized
and Delocalized Chemical Bonding Theory. J.
Comput. Chem..

[52] Glendening E. D., Landis C. R., Weinhold F. (2013). NBO 6.0: Natural Bond Orbital Analysis
Program. J. Comput. Chem..

[53] Glendening E. D., Weinhold F. (1998). Natural Resonance Theory: I. General Formalism. J. Comput. Chem..

[54] Glendening E. D., Weinhold F. (1998). Natural Resonance Theory:
II. Natural Bond Order and
Valency. J. Comput. Chem..

[55] Glendening E. D., Badenhoop J. K., Weinhold F. (1998). Natural Resonance Theory: III. Chemical
Applications. J. Comput. Chem..

[56] Pegamoid, Graphical Program, V. 2.12.4; https://github.com/Jellby/Pegamoid (accessed 08 December 2026).

[57] ChemCraft, Graphical Program, V. B814bt; https://www.chemcraftprog.com (accessed 08 December 2026).

[58] Molden, Graphical Software, V. 6.9, G.Schaftenaar, Theor. & Comp. Chem. Group, the Netherlands; https://www.theochem.ru.nl/molden (accessed 08 December 2026).

[59] Béthegnies A., Kirkina V. A., Filippov O. A., Daran J.-C., Belkova N. V., Shubina E., Poli R. (2011). Coordination and Organometallic Chemistry
of Relevance to the Rhodium-Based Catalyst for Ethylene Hydroamination. Inorg. Chem..

[60] Dachs A., Osuna S., Roglans A., Solà M. (2010). Density Functional
Study of the [2+2+2] Cyclotrimerization of Acetylene Catalyzed by
Wilkinson’s Catalyst, RhCl­(PPh_3_)_3_. Organometallics.

[61] Arévalo A., Juárez-Francisco E., Roa D. A., Flores-Alamo M., García J. J. (2024). New Mononuclear Cu­(i) Compounds: Synthesis, Characterization,
and Application to the Electroreduction of CO_2_. Catal. Sci. Technol..

[62] Kiefer J., Obert K., Himmler S., Schulz P. S., Wasserscheid P., Leipertz A. (2008). Infrared Spectroscopy of a Wilkinson Catalyst in a
Room-Temperature Ionic Liquid. ChemPhyschem.

[63] Perea-Buceta J. E., Fernández I., Heikkinen S., Axenov K., King A. W. T., Niemi T., Nieger M., Leskelä M., Repo T. (2015). Diverting Hydrogenations
with Wilkinson’s Catalyst towards
Highly Reactive Rhodium­(I) Species. Angew. Chem.
Int. Ed..

[64] Brookhart M., Green M. L. H., Parkin G. (2007). Agostic Interactions in Transition
Metal Compounds. Proc. Natl. Acad. Sci. U.S.A..

[65] Reinholdt A., Vibenholt J. E., Morsing T. J., Schau-Magnussen M., Reeler N. E. A., Bendix J. (2015). Carbide Complexes
as π-Acceptor
Ligands. Chem. Sci..

[66] Collins S., Linnolahti M. (2022). Activation of Substituted Metallocene Catalysts Using
Methylaluminoxane. ChemCatchem.

[67] Meelua W., Keawkla N., Oláh J., Jitonnom J. (2020). DFT Study of Formation
and Properties of Dinuclear Zirconocene Cations: Effects of Ligand
Structure, Solvent, and Metal on the Dimerization Process. J. Organomet. Chem..

