# Supplementary material for: Multicenter Bonding in Wilkinson’s Catalyst: A Theoretical Study from a Distorted Monomer to a Localized Dimer
Source: J Phys Chem A. 2026 Sep 28;130(40):7915–27. doi: 10.1021/acs.jpca.6c04219 (PMC13656780; doi:10.1021/acs.jpca.6c04219)
Supplement: Supplementary file 1 [file jp6c04219_si_001.pdf]

Multicenter Bonding in Wilkinson's Catalyst: A Theoretical Study from a Distorted Monomer to a Localized Dimer

Dr. rer. nat. Aleksandr Zaichenko[a.b.c]. Prof. Dr. Holger F. Bettinger[d]. Prof. Dr. Ivana Fleischer[d]. Prof. Dr. Doreen Mollenhauer[*.a.b.c]

[a] Helmholtz Institute for Polymers in Energy Applications Jena (HIPOLE Jena). Lessingstrasse 12-14. 07743 Jena. Germany

[b] Helmholtz-Zentrum Berlin für Materialien und Energie GmbH (HZB). Hahn-Meitner-Platz 1. 14109 Berlin. Germany

[c] Institute for Technical and Environmental Chemistry. Friedrich Schiller University Jena. Philosophenweg 7a. 07743 Jena. Germany

[d] Institut für Organische Chemie. Auf der Morgenstelle 18. 72076 Tübingen. Germany

doreen.mollenhauer@helmholtz-berlin.de

STRUCRURAL DATA

TPSSh-D4/[def2-SVP (non-Rh) + def2-TZVPP/def2-ECP (Rh)]

Table SI1: a) Wilkinson catalyst monomer structure

Rh -0.032627 0.414115 -0.386461
C -1.782922 -2.539362 0.420290
C -2.628424 -2.437830 1.539026
C -3.863657 -3.090787 1.572002
C -4.294391 -3.841780 0.476612
C -3.487187 -3.911446 -0.661800
C -2.249083 -3.267027 -0.691361
C 0.608743 -2.659057 -1.082766
C 0.954339 -4.015934 -0.964331
C 1.435521 -4.718224 -2.071506
C 1.554228 -4.079280 -3.312038
C 1.188740 -2.736959 -3.442105
C 0.722850 -2.028638 -2.331242
C 0.806756 -2.355052 1.775097
C 0.284935 -2.348444 3.079788
C 1.069537 -2.738548 4.168651
C 2.397498 -3.130854 3.980379
C 2.936068 -3.124323 2.691102
C 2.149489 -2.737032 1.605491
P -0.126055 -1.700587 0.315928
C 3.124533 0.256055 1.283573
C 2.345467 0.344105 2.446813
C 2.931143 0.202039 3.705103
C 4.305010 -0.038944 3.810583
C 5.090388 -0.116542 2.655398
C 4.506592 0.043101 1.395115
C 2.761277 2.410103 -0.408991

C 4.011268 2.811733 -0.899913
C 4.374294 4.162526 -0.871977
C 3.497577 5.115290 -0.342288
C 2.253737 4.714664 0.159907
C 1.885952 3.368644 0.122846
C 3.231876 -0.169117 -1.629425
C 3.913428 -1.383700 -1.455061
C 4.496671 -2.032775 -2.546638
C 4.405503 -1.475845 -3.825166
C 3.717431 -0.270781 -4.009158
C 3.126666 0.376787 -2.922554
P 2.270997 0.637302 -0.298400
Cl 0.010595 2.001701 -2.167581
P -2.247843 0.851843 -0.107361
C -2.827906 0.822668 1.636450
C -4.139969 1.159531 2.007952
C -4.503635 1.181718 3.357184
C -3.554554 0.902522 4.348613
C -2.233971 0.615884 3.987507
C -1.875446 0.580014 2.636834
C -3.392607 -0.131903 -1.144866
C -2.937071 -0.351550 -2.458826
C -3.719812 -1.070151 -3.363667
C -4.957477 -1.587149 -2.962577
C -5.404591 -1.383335 -1.654406
C -4.627611 -0.656785 -0.746188
C -2.686749 2.589740 -0.519795
C -2.118867 3.596996 0.278204
C -2.366179 4.940577 -0.000296
C -3.184035 5.292343 -1.082199
C -3.752747 4.292906 -1.876111
C -3.505154 2.943255 -1.598500
H -2.349429 -1.835626 2.398851
H -4.491294 -3.001208 2.461889
H -5.260098 -4.352131 0.501499
H -3.821196 -4.468214 -1.539981
H -1.640982 -3.335878 -1.592451
H 0.840773 -4.528171 -0.006246
H 1.710363 -5.770951 -1.968444
H 1.929991 -4.631676 -4.176879
H 1.277219 -2.232069 -4.405848
H 0.443414 -0.972560 -2.418991
H -0.740145 -2.038542 3.268975
H 0.635780 -2.731658 5.171637
H 3.010808 -3.430148 4.833316
H 3.976336 -3.413284 2.526728
H 2.584429 -2.735983 0.607064
H 1.272301 0.524162 2.340233
H 2.314076 0.267321 4.603619
H 4.765638 -0.162305 4.793735
H 6.166230 -0.291052 2.735458
H 5.134055 0.011237 0.502156
H 4.702276 2.073597 -1.313263
H 5.345976 4.470421 -1.266035
H 3.782297 6.170174 -0.323511
H 1.561843 5.455209 0.568394
H 0.907715 3.048612 0.488235
H 3.996481 -1.828816 -0.463430
H 5.020968 -2.978959 -2.394767
H 4.862679 -1.983044 -4.678191

H 3.633171 0.165559 -5.007478
H 2.558195 1.297900 -3.070845
H -4.875901 1.426923 1.246930
H -5.529373 1.432524 3.637893
H -3.842058 0.928325 5.402466
H -1.481989 0.423986 4.756817
H -0.849514 0.361368 2.323589
H -1.966942 0.057149 -2.757918
H -3.359904 -1.231938 -4.382562
H -5.568423 -2.156676 -3.667272
H -6.360948 -1.801074 -1.331108
H -4.980416 -0.526941 0.276643
H -1.478996 3.322381 1.121013
H -1.918858 5.717223 0.624965
H -3.376519 6.344849 -1.303899
H -4.391870 4.561728 -2.720785
H -3.946956 2.168911 -2.228090

b) Wilkinson catalyst monomer structure in vacuum

Rh	-0.0371979	0.3974212	-0.3851947
C	-1.7822696	-2.5517538	0.4217455
C	-2.6328001	-2.4548407	1.5368407
C	-3.8677456	-3.1079207	1.5617486
C	-4.2939754	-3.8532335	0.4611996
C	-3.4828533	-3.9162678	-0.6742487
C	-2.2443656	-3.2728290	-0.6955931
C	0.6164424	-2.6696999	-1.0698231
C	0.9703736	-4.0238405	-0.9469469
C	1.4589699	-4.7257621	-2.0507684
C	1.5767866	-4.0894641	-3.2922964
C	1.2027444	-2.7502877	-3.4268748
C	0.7291680	-2.0415498	-2.3196951
C	0.8055131	-2.3701027	1.7863104
C	0.2781939	-2.3852680	3.0883991
C	1.0646604	-2.7725489	4.1767305
C	2.3990514	-3.1418852	3.9909474
C	2.9423888	-3.1153873	2.7042502
C	2.1547823	-2.7296513	1.6194007
P	-0.1254012	-1.7121123	0.3268317
C	3.1177649	0.2645389	1.2781755
C	2.3364000	0.3483884	2.4401002
C	2.9193668	0.2030735	3.6988853
C	4.2926673	-0.0373725	3.8069175
C	5.0803801	-0.1111049	2.6535953
C	4.4993909	0.0517841	1.3928220
C	2.7441323	2.4154606	-0.4286392
C	4.0087065	2.8104695	-0.8867490
C	4.3668947	4.1617491	-0.8765717
C	3.4697918	5.1225032	-0.3991028
C	2.2111354	4.7293311	0.0679077
C	1.8483046	3.3818637	0.0502643
C	3.2288997	-0.1626923	-1.6334527
C	3.9138279	-1.3752946	-1.4590543
C	4.5024520	-2.0207523	-2.5494474
C	4.4125118	-1.4625291	-3.8271455
C	3.7201432	-0.2603749	-4.0111914
C	3.1246032	0.3847951	-2.9259968
P	2.2630890	0.6419623	-0.3045678
Cl	-0.0072858	1.9409009	-2.1967396

P	-2.2452834	0.8498925	-0.1012089
C	-2.8328066	0.8168089	1.6409044
C	-4.1409315	1.1737905	2.0069413
C	-4.5120335	1.1959936	3.3537672
C	-3.5737177	0.8966239	4.3489644
C	-2.2565204	0.5897065	3.9939067
C	-1.8905378	0.5538632	2.6453974
C	-3.4010233	-0.1200651	-1.1398781
C	-2.9595207	-0.3249680	-2.4611560
C	-3.7505183	-1.0388454	-3.3622854
C	-4.9806591	-1.5654519	-2.9517646
C	-5.4127299	-1.3765456	-1.6368153
C	-4.6281837	-0.6552826	-0.7313550
C	-2.6643283	2.5944995	-0.5034868
C	-2.1101027	3.5890855	0.3192498
C	-2.3369907	4.9369476	0.0455337
C	-3.1201057	5.3054510	-1.0552442
C	-3.6753049	4.3186863	-1.8730758
C	-3.4485256	2.9650244	-1.6014360
H	-2.3575308	-1.8553433	2.3997799
H	-4.4985341	-3.0226578	2.4498368
H	-5.2593112	-4.3645859	0.4799424
H	-3.8134511	-4.4678810	-1.5569141
H	-1.6320622	-3.3379320	-1.5941298
H	0.8572579	-4.5329167	0.0128266
H	1.7399484	-5.7765151	-1.9443351
H	1.9584617	-4.6415612	-4.1547297
H	1.2907022	-2.2465319	-4.3911695
H	0.4425309	-0.9877454	-2.4133295
H	-0.7541151	-2.0980240	3.2740055
H	0.6260827	-2.7846941	5.1776610
H	3.0132768	-3.4401301	4.8435515
H	3.9877108	-3.3864404	2.5418065
H	2.5928362	-2.7120714	0.6224756
H	1.2632535	0.5274844	2.3305196
H	2.3000355	0.2651896	4.5960505
H	4.7512563	-0.1628359	4.7907504
H	6.1562838	-0.2842791	2.7358473
H	5.1277743	0.0224509	0.5004492
H	4.7132708	2.0651446	-1.2627455
H	5.3501878	4.4645268	-1.2449037
H	3.7504849	6.1786421	-0.3955891
H	1.5013966	5.4760840	0.4316719
H	0.8579179	3.0679530	0.3856754
H	3.9945236	-1.8211465	-0.4674599
H	5.0294392	-2.9654406	-2.3976261
H	4.8746621	-1.9664774	-4.6794324
H	3.6367936	0.1769518	-5.0091337
H	2.5524740	1.3037721	-3.0739514
H	-4.8655781	1.4583885	1.2412176
H	-5.5346521	1.4638475	3.6301844
H	-3.8666525	0.9235918	5.4013264
H	-1.5112703	0.3827294	4.7659265
H	-0.8662333	0.3210031	2.3371263
H	-1.9954363	0.0928443	-2.7683042
H	-3.4024271	-1.1886122	-4.3870625
H	-5.5977971	-2.1303305	-3.6548056
H	-6.3631667	-1.8018511	-1.3059775
H	-4.9674621	-0.5377082	0.2976117
H	-1.4978793	3.3006916	1.1778435

H	-1.9008578	5.7038666	0.6904160
H	-3.2958500	6.3615258	-1.2735447
H	-4.2864717	4.6009993	-2.7337661
H	-3.8778960	2.2001681	-2.2505240

c) Wilkinson catalyst monomer planar conformer structure

Rh	0.0602257	-0.7245836	-0.0408695
Cl	-0.0037289	-3.0966242	-0.1452907
P	-0.0120201	1.4997614	0.0392389
P	2.3516271	-0.8419073	0.0785492
P	-2.2573775	-0.9349712	-0.2218045
C	1.4625694	2.5693763	0.3782580
C	1.7073697	3.1068161	1.6514478
C	2.8210817	3.9206705	1.8734568
C	3.7088856	4.2036277	0.8311477
C	3.4762947	3.6661814	-0.4385551
C	2.3583726	2.8601115	-0.6641554
C	-0.5662515	2.2857174	-1.5359713
C	-0.6020754	3.6772186	-1.7307761
C	-1.0080138	4.2068398	-2.9584623
C	-1.3550556	3.3548506	-4.0148267
C	-1.2854062	1.9690505	-3.8420304
C	-0.8914929	1.4431910	-2.6083923
C	-1.1297326	2.1074393	1.3756551
C	-1.8946605	3.2795886	1.3228667
C	-2.6237045	3.6922528	2.4417514
C	-2.5892751	2.9473240	3.6250558
C	-1.8385373	1.7690072	3.6805543
C	-1.1203942	1.3505774	2.5582874
C	3.2252936	-0.2947639	-1.4409270
C	4.6211169	-0.3714920	-1.5863940
C	5.2240672	0.0248054	-2.7827096
C	4.4391032	0.4720147	-3.8535247
C	3.0465720	0.5064073	-3.7308036
C	2.4455183	0.1204903	-2.5291455
C	3.0609408	0.0690730	1.5056597
C	2.3470152	-0.0668411	2.7090034
C	2.8180076	0.5189700	3.8853386
C	4.0071656	1.2561951	3.8676092
C	4.7132065	1.4062012	2.6703748
C	4.2458622	0.8143723	1.4928851
C	3.1317479	-2.4984852	0.2889599
C	3.1419212	-3.3656362	-0.8174469
C	3.7079212	-4.6359774	-0.7128666
C	4.2610552	-5.0620233	0.5011883
C	4.2469741	-4.2072736	1.6062869
C	3.6852053	-2.9290899	1.5017979
C	-2.7909398	-1.5329216	-1.8825000
C	-4.1298079	-1.4601364	-2.3060971
C	-4.4928437	-1.8959432	-3.5837119
C	-3.5218770	-2.3988227	-4.4566809
C	-2.1868224	-2.4683856	-4.0446558
C	-1.8230261	-2.0400279	-2.7649738
C	-2.8943838	-2.1663034	0.9876016
C	-2.2091342	-2.2907615	2.2057524
C	-2.6906333	-3.1392819	3.2048732
C	-3.8534460	-3.8869165	2.9866765
C	-4.5301812	-3.7802009	1.7669334
C	-4.0563804	-2.9188916	0.7720124

C	-3.4623824	0.4410491	0.0497213
C	-4.1093587	0.5909408	1.2870018
C	-5.0048685	1.6423107	1.4966462
C	-5.2669477	2.5614112	0.4762993
C	-4.6244544	2.4227352	-0.7574325
C	-3.7311591	1.3703782	-0.9695048
H	1.0241082	2.8965738	2.4747887
H	2.9957266	4.3324307	2.8699820
H	4.5790421	4.8405349	1.0080413
H	4.1611586	3.8808091	-1.2625480
H	2.1756355	2.4719192	-1.6658969
H	-0.2900382	4.3518482	-0.9308873
H	-1.0415565	5.2903320	-3.0965911
H	-1.6649929	3.7739796	-4.9752336
H	-1.5397476	1.2941083	-4.6626923
H	-0.8364756	0.3620750	-2.4558395
H	-1.9444535	3.8667049	0.4060317
H	-3.2297660	4.5994546	2.3845960
H	-3.1598775	3.2771984	4.4966298
H	-1.8188397	1.1698541	4.5941348
H	-0.5420496	0.4239860	2.5757902
H	5.2372313	-0.7621299	-0.7737356
H	6.3106150	-0.0279283	-2.8859707
H	4.9150861	0.7754718	-4.7891979
H	2.4258301	0.8315659	-4.5691258
H	1.3590982	0.1303739	-2.4126522
H	1.4175187	-0.6436265	2.7059560
H	2.2548917	0.4037391	4.8145613
H	4.3775125	1.7222437	4.7839198
H	5.6311601	1.9980370	2.6473718
H	4.7973613	0.9589413	0.5640044
H	2.7056812	-3.0417338	-1.7641673
H	3.7105712	-5.3007444	-1.5800504
H	4.7018367	-6.0585753	0.5833570
H	4.6793055	-4.5304542	2.5565039
H	3.6875918	-2.2685977	2.3703693
H	-4.8927660	-1.0447869	-1.6447052
H	-5.5372189	-1.8357948	-3.8995057
H	-3.8060582	-2.7331656	-5.4575453
H	-1.4226647	-2.8587832	-4.7211235
H	-0.7854751	-2.1023403	-2.4283281
H	-1.2807015	-1.7325441	2.3462790
H	-2.1483150	-3.2297426	4.1491456
H	-4.2264414	-4.5600039	3.7625813
H	-5.4309336	-4.3718937	1.5862492
H	-4.5910763	-2.8479253	-0.1765604
H	-3.9189993	-0.1203912	2.0914838
H	-5.4988920	1.7422011	2.4656904
H	-5.9694481	3.3818076	0.6423286
H	-4.8218715	3.1316107	-1.5652587
H	-3.2578680	1.2656814	-1.9447158

Table SI2: $[Rh(PPh_3)_2Cl]$ fragment

a) *cis* reaction path

Rh	0.0104064	0.2820681	-0.0935437
C	-1.3288468	-2.4146751	0.1375873
C	-2.2178395	-1.6300900	0.8991341

C	-3.5845436	-1.9238621	0.9138227
C	-4.0713942	-3.0042865	0.1715917
C	-3.1920941	-3.7868906	-0.5877537
C	-1.8264974	-3.4930688	-0.6116117
C	1.1040073	-2.7345383	-1.3761599
C	1.6630686	-4.0216291	-1.3445865
C	2.1087111	-4.6179865	-2.5285615
C	1.9855733	-3.9407224	-3.7465056
C	1.4131812	-2.6642715	-3.7833397
C	0.9778012	-2.0620072	-2.6025989
C	1.2145677	-2.6881824	1.5272510
C	0.4680049	-2.9560057	2.6857401
C	1.0949968	-3.4571846	3.8300827
C	2.4730886	-3.6947543	3.8319205
C	3.2234509	-3.4328269	2.6810621
C	2.5985707	-2.9313218	1.5386508
P	0.4242980	-1.8699815	0.0870700
C	2.9891866	0.3327466	1.5179450
C	2.2176446	0.0778310	2.6625890
C	2.8348445	-0.2273565	3.8768227
C	4.2297125	-0.2895033	3.9538023
C	5.0055390	-0.0237441	2.8196705
C	4.3899647	0.2997616	1.6072470
C	2.4844170	2.6398295	-0.0141871
C	3.7059780	3.1118293	-0.5246595
C	4.0345128	4.4660638	-0.4190626
C	3.1508174	5.3564907	0.2012668
C	1.9359870	4.8885779	0.7130480
C	1.6015101	3.5355229	0.6065296
C	3.0523112	0.2125545	-1.4362119
C	3.9034045	-0.8998221	-1.3781627
C	4.4967956	-1.4003925	-2.5413054
C	4.2504369	-0.7893344	-3.7727348
C	3.3997237	0.3214418	-3.8403589
C	2.7961472	0.8132718	-2.6829972
P	2.0953017	0.8382141	-0.0049206
Cl	-1.2988257	2.1608324	-0.4722012
H	-1.8305497	-0.7858748	1.4827879
H	-4.2663244	-1.3053467	1.5014428
H	-5.1391212	-3.2352813	0.1792404
H	-3.5747024	-4.6282106	-1.1704529
H	-1.1471216	-4.0961786	-1.2172095
H	1.7492351	-4.5616374	-0.3995919
H	2.5487680	-5.6177915	-2.4995462
H	2.3361073	-4.4096683	-4.6692184
H	1.3168400	-2.1320193	-4.7317706
H	0.5443137	-1.0570610	-2.6083561
H	-0.6085434	-2.7748885	2.6972570
H	0.5015062	-3.6647647	4.7236971
H	2.9618730	-4.0824993	4.7286777
H	4.3008130	-3.6113778	2.6737573
H	3.1931018	-2.7310295	0.6475685
H	1.1278448	0.1140245	2.5767534
H	2.2254382	-0.4311700	4.7595467
H	4.7151847	-0.5416077	4.8996832
H	6.0959610	-0.0590488	2.8811348
H	5.0054954	0.5321050	0.7356263
H	4.4017642	2.4247044	-1.0110073
H	4.9831805	4.8263308	-0.8244454
H	3.4078808	6.4155161	0.2813327

H	1.2384832	5.5805036	1.1909399
H	0.6403765	3.1736569	0.9758703
H	4.1130895	-1.3778893	-0.4214599
H	5.1511012	-2.2727383	-2.4812377
H	4.7140343	-1.1806762	-4.6812383
H	3.1990165	0.8010109	-4.8012574
H	2.1148649	1.6659854	-2.7432243

b) *trans* reaction path

Rh	0.1642144	1.4112483	-0.3480302
C	2.4925364	0.6177322	0.9174294
C	1.1980164	0.6926758	1.4941535
C	0.9021702	-0.0714863	2.6316432
C	1.8734555	-0.9461626	3.1336142
C	3.1245145	-1.0689033	2.5144754
C	3.4377681	-0.2926417	1.3891631
C	3.2432820	3.2233728	-0.2301489
C	4.1196141	3.4000569	0.8515587
C	4.7124412	4.6482184	1.0709127
C	4.4344092	5.7177615	0.2132283
C	3.5543535	5.5445382	-0.8629129
C	2.9531968	4.3036090	-1.0824331
C	3.4488172	0.8382394	-1.8613779
C	2.8087536	-0.0223012	-2.7683540
C	3.5615100	-0.6964517	-3.7339413
C	4.9470671	-0.5104961	-3.7995418
C	5.5838758	0.3577178	-2.9042103
C	4.8374076	1.0367293	-1.9374771
P	2.4268310	1.6354734	-0.5856368
Cl	-0.3440838	1.8598324	-2.5847853
P	-2.0684663	1.1288806	0.1461763
C	-2.3989637	0.7460742	1.9125945
C	-2.4352596	1.7875541	2.8542003
C	-2.5410972	1.5025954	4.2189855
C	-2.6187693	0.1757036	4.6571236
C	-2.5839826	-0.8666822	3.7235940
C	-2.4655712	-0.5849505	2.3597156
C	-3.0444174	-0.1534091	-0.7368730
C	-2.5499662	-0.6690595	-1.9454966
C	-3.2802833	-1.6310251	-2.6506998
C	-4.5050875	-2.0896086	-2.1561664
C	-5.0013715	-1.5839835	-0.9489420
C	-4.2755131	-0.6227081	-0.2412547
C	-2.9907789	2.6994347	-0.1112319
C	-2.2349431	3.8726350	-0.2642909
C	-2.8724391	5.1080322	-0.4151175
C	-4.2689582	5.1775568	-0.4242229
C	-5.0274563	4.0089424	-0.2821832
C	-4.3925074	2.7744221	-0.1251994
H	0.5421596	1.6699599	1.3402492
H	-0.0711130	0.0072240	3.1143857
H	1.6475258	-1.5481443	4.0171621
H	3.8611813	-1.7705679	2.9126611
H	4.4010745	-0.4021258	0.8852535
H	4.3329493	2.5654990	1.5236102
H	5.3934740	4.7846694	1.9143594
H	4.8990257	6.6912909	0.3867283
H	3.3293590	6.3822113	-1.5271310
H	2.2486290	4.1676641	-1.9074395

H	1.7223776	-0.1318661	-2.7233908
H	3.0634907	-1.3614087	-4.4433059
H	5.5328685	-1.0360909	-4.5575517
H	6.6643719	0.5091359	-2.9622518
H	5.3317381	1.7246363	-1.2470350
H	-2.3795572	2.8259031	2.5184913
H	-2.5677305	2.3210838	4.9422160
H	-2.7064574	-0.0453818	5.7234263
H	-2.6405575	-1.9052307	4.0582542
H	-2.4151613	-1.4055122	1.6410542
H	-1.6023709	-0.2919529	-2.3361291
H	-2.8869711	-2.0235207	-3.5914193
H	-5.0724852	-2.8433877	-2.7075453
H	-5.9551679	-1.9418277	-0.5537914
H	-4.6638948	-0.2522464	0.7099470
H	-1.1430227	3.7955179	-0.2775436
H	-2.2758754	6.0154361	-0.5352106
H	-4.7690406	6.1411611	-0.5489050
H	-6.1189069	4.0588882	-0.2970061
H	-4.9952339	1.8704978	-0.0233191

Table SI3: SPC structure

Rh -0.004995 0.585239 -0.229289
H -1.257027 -1.941398 1.229821
Cl 0.073527 2.748180 -1.194413
H -2.872065 1.048630 -1.530002
H 3.128775 -0.340442 0.180556
H -2.780443 1.995210 0.396206
P -2.264783 0.855019 -0.265430
H 0.234851 -2.567419 -0.155747
P -0.061197 -1.433344 0.653677
H 2.883114 1.785833 0.311485
H 2.855011 0.812550 -1.604769
H -3.169798 -0.110534 0.250634
P 2.273183 0.676400 -0.321088
H 0.810801 -1.767924 1.727078

Table SI4: DSPC structure

Rh	-0.032642	0.414014	-0.386537
Cl	0.010622	2.001609	-2.167751
P	-0.126039	-1.700625	0.315947
P	2.271003	0.637325	-0.298473
P	-2.247813	0.851711	-0.107399
H	-1.386068	-2.338884	0.395605
H	2.648205	2.001666	-0.383474
H	-3.132190	0.091740	-0.907746
H	-2.586794	2.192427	-0.424905
H	0.437695	-2.437261	-0.757910
H	2.928477	0.342701	0.920434
H	3.013459	0.013119	-1.327614
H	-2.693487	0.828727	1.232871
H	0.588116	-2.202559	1.433683

Table SI5: Dimer structure

Rh	-1.531500	0.031867	0.748171
C	-3.142905	1.874073	-1.725215

C	-2.594748	0.930864	-2.603550
C	-2.750562	1.066131	-3.986540
C	-3.441636	2.166356	-4.503524
C	-3.961555	3.134625	-3.634450
C	-3.810413	2.993298	-2.252384
C	-4.583457	1.479191	0.811205
C	-5.778111	1.797487	0.154553
C	-6.996635	1.712450	0.836076
C	-7.031581	1.320013	2.177197
C	-5.842079	0.988254	2.835355
C	-4.626694	1.057563	2.152030
C	-2.434682	3.337397	0.601622
C	-2.973556	3.948294	1.742409
C	-2.508795	5.201242	2.158156
C	-1.503873	5.855275	1.440301
C	-0.967639	5.252655	0.296953
C	-1.427991	4.003156	-0.116488
P	-2.900614	1.627835	0.081125
Cl	-0.006577	-1.608874	1.688709
Cl	0.006370	1.607714	1.689435
P	-2.775741	-1.657232	0.012897
C	-2.134126	-2.489538	-1.499090
C	-2.887519	-3.449180	-2.198513
C	-2.367298	-4.060277	-3.341843
C	-1.092144	-3.711172	-3.801581
C	-0.340327	-2.753306	-3.114903
C	-0.856625	-2.144603	-1.965538
C	-4.567537	-1.495419	-0.406728
C	-4.965671	-1.109374	-1.697293
C	-6.317885	-0.949669	-2.009043
C	-7.294232	-1.175577	-1.035055
C	-6.907355	-1.557778	0.253066
C	-5.555548	-1.715684	0.566039
C	-2.813375	-3.000403	1.277917
C	-2.624735	-4.355394	0.978187
C	-2.636989	-5.311719	1.999004
C	-2.846771	-4.924654	3.325316
C	-3.031087	-3.571411	3.632440
C	-3.001567	-2.614892	2.616506
H	-2.028453	0.096315	-2.186673
H	-2.319188	0.318448	-4.656405
H	-3.562669	2.280933	-5.583512
H	-4.483051	4.006524	-4.036772
H	-4.197841	3.764460	-1.582562
H	-5.767970	2.094289	-0.894872
H	-7.925137	1.948430	0.310821
H	-7.986389	1.258892	2.705147
H	-5.861544	0.666947	3.879573
H	-3.689135	0.782824	2.645478
H	-3.758460	3.450617	2.314141
H	-2.937725	5.666066	3.049395
H	-1.139084	6.830917	1.770617
H	-0.180040	5.752155	-0.271746
H	-0.990466	3.537830	-0.998443
H	-3.889534	-3.713363	-1.853286
H	-2.960277	-4.805819	-3.877321
H	-0.685329	-4.183879	-4.699004
H	0.653595	-2.480598	-3.471071
H	-0.275597	-1.395475	-1.412779
H	-4.220552	-0.948333	-2.475184

H	-6.605812	-0.652333	-3.020392
H	-8.352714	-1.053828	-1.277475
H	-7.661548	-1.733205	1.023297
H	-5.271950	-2.022418	1.573279
H	-2.443911	-4.670274	-0.050085
H	-2.473053	-6.364178	1.754720
H	-2.853536	-5.673631	4.121047
H	-3.178377	-3.258111	4.668812
H	-3.092439	-1.552524	2.855964
Rh	1.531857	-0.032632	0.749050
C	2.431686	-3.338029	0.599803
C	1.430641	-4.005354	-0.124641
C	0.966433	-5.253535	0.288569
C	1.493034	-5.853157	1.437904
C	2.492316	-5.197469	2.162158
C	2.960919	-3.945916	1.746734
C	3.144963	-1.872683	-1.724885
C	3.812877	-2.991234	-2.252910
C	3.966035	-3.130297	-3.634960
C	3.447040	-2.160831	-4.503239
C	2.754096	-1.062121	-3.985546
C	2.596639	-0.928929	-2.602547
C	4.582611	-1.482229	0.813865
C	5.777869	-1.801327	0.158683
C	6.995628	-1.717059	0.841665
C	7.029200	-1.325064	2.182950
C	5.839085	-0.992973	2.839809
C	4.624521	-1.061073	2.154892
P	2.900535	-1.628659	0.081415
P	2.777372	1.656352	0.015517
C	2.137201	2.489344	-1.496703
C	0.859176	2.146094	-1.963039
C	0.343443	2.755612	-3.112222
C	1.096423	3.712548	-3.798969
C	2.372286	4.059573	-3.339662
C	2.891824	3.447853	-2.196336
C	4.569473	1.493835	-0.402179
C	5.556474	1.712862	0.571883
C	6.908508	1.553677	0.260551
C	7.296645	1.172148	-1.027388
C	6.321306	0.947608	-2.002703
C	4.968825	1.107836	-1.692394
C	2.814267	2.999187	1.280881
C	3.000011	2.613292	2.619691
C	3.029158	3.569684	3.635769
C	2.846980	4.923179	3.328496
C	2.639467	5.310637	2.001932
C	2.627564	4.354425	0.981006
H	1.000317	-3.542270	-1.011284
H	0.183245	-5.754287	-0.285117
H	2.913835	-5.660028	3.058099
H	3.741202	-3.446844	2.323567
H	4.199369	-3.763461	-1.583791
H	4.488724	-4.001188	-4.037925
H	3.569850	-2.273530	-5.583226
H	2.323142	-0.313666	-4.654821
H	2.029148	-0.095506	-2.185038
H	5.768891	-2.097783	-0.890845
H	7.924615	-1.953412	0.317434
H	7.983413	-1.264611	2.712053

H	5.857443	-0.672231	3.884222
H	3.686585	-0.785459	2.647151
H	0.277264	1.397619	-1.410297
H	2.966221	4.804271	-3.875247
H	3.894229	3.710704	-1.851231
H	5.271852	2.019186	1.578967
H	7.661926	1.728222	1.031737
H	8.355340	1.049944	-1.268658
H	6.610222	0.650854	-3.013939
H	4.224480	0.947625	-2.471192
H	3.089278	1.550778	2.859128
H	3.174563	3.256133	4.672331
H	2.853507	5.672082	4.124297
H	2.477201	6.363327	1.757527
H	2.448698	4.669615	-0.047530
H	0.689924	4.186194	-4.696041
H	-0.650958	2.484282	-3.468144
H	1.125025	-6.827640	1.768064

IR Spectrum

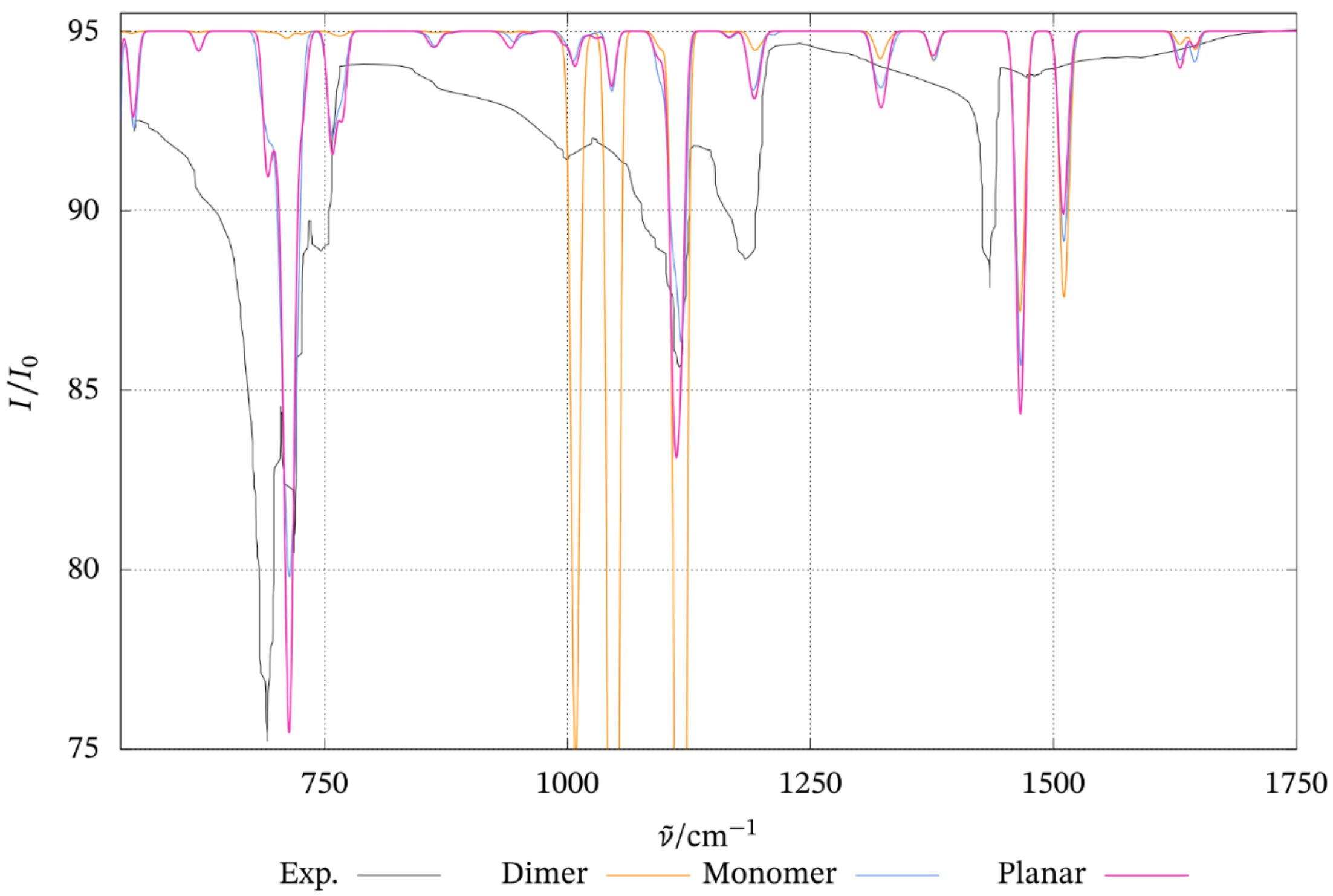

Figure SI1. Comparison of the experimental solid-state [44] and calculated TPSSh-D4/[def2-SVP (non-Rh) + def2-TZVPP/def2-ECP (Rh)] (COSMO. toluene) spectra of Wilkinson's catalyst in its monomeric and dimeric forms.

ENERGY ESTIMATION

Table SI6: Energy data for Wilkinson monomer dimerization

	Dimer	Monomer	Mon_planar	Frag trans	Frag cis	Ligand
E_el	-5285.10007	-3678.41944	-3678.43053	-2642.47106	-2642.49503	-1035.85686
H/kJ/mol	3074.29000	2298.55000	2298.75000	1530.75000	1535.78000	756.07000
	1.17094	0.87547	0.87555	0.58303	0.58495	0.28797
Bsse	0.01086			0.01136	0.00911	
S/kJ/mol/K	1.64012	1.29692	1.29836	1.02906	0.97398	0.56083
ST	0.18625	0.14728	0.14744	0.11686	0.11060	0.06369
H0						
Ha	-0.09810		0.08670	0.07568	0.05588	
eV	-2.66955		2.35916	2.05945	1.52068	
kJ/mol	-257.57243		227.62409	198.70694	146.72287	51.98407
G0						
Ha	-0.06315		0.05359	0.04241	0.02887	
eV	-1.71829		1.45830	1.15414	0.78557	
kJ/mol	-165.78990	0.00000	140.70439	111.35790	75.79594	35.56196
Relaxation Energy el				0.03437	0.02910	
eV				0.93520	0.79194	
kJ/mol				90.23297	76.41037	

HOMO-LUMO ENERGIES

Table SI7: HOMO-LUMO energies at different levels of theory (HF calculation parameters are the same as those used for the CCSD) eV

eV	TPSSh	r2SCAN	HF
SPC	3.77	3.04	6.79
DSPC	3.06	2.35	5.94
Monomer	2.84	2.11	-
Monimer(pl)	2.99	2.28	
Dimer	2.67	1.99	-
Fragment-trans	2.74	2.05	
Fragment-cis	2.52	1.85	

RESONANCES

Table SI8: Contribution of resonance structures of systems at the X2C-r2SCAN/[x2c-def2-TZVPall (non-Rh) + x2c-def2-TZVPPall (Rh)] level of theory

Resonances	SPC	DSPC	Wilkinson	Planar	Dimer
a	25.88	29.71	28.48	27.24	35.62
b	25.77	22.1	24.76	25.16	17.79
c	18.14	17.12	13.07	12.98	12.05
d	18.85	20.61	18.25	19.46	smaller

Table SI9: Contribution of resonance structures of small model systems at the X2C-CCSD/[x2c-def2-SVP (non-Rh) + x2c-def2-TZVPPall (Rh)] level of theory

Resonances	SPC	DSPC
a	24.91	22.58
b	24.3	26.55
c	19.05	15.51
d	19.26	23.69

BONDING

Table SI10: Bond parameters of systems calculated at the single point X2C-TPSSh/[x2c-def2-TZVPall (non-Rh) + x2c-def2-TZVPPall (Rh)] level of theory

	DSPC				
	Rh-P2	Rh-P1	Rh-P3	Rh-Cl	
MBO	0.8104	0.875	1.0773	0.7976	
WBI	0.5025	0.548	0.6477	0.4113	
NLMO	0.3252	0.5352	0.5695	0.2926	
	Rh	Rh-Cl	Rh-P3	Rh-P1	Rh-P2
total	4.0985	0.3893	0.5122	0.4707	0.4309
covalent		0.1772	0.2754	0.2645	0.2228
ionic		0.2121	0.2368	0.2062	0.2081
	SPC				
	Rh-P2	Rh-P1	Rh-P3	Rh-Cl	
MBO	0.8221	0.8305	1.0915	0.7432	
WBI	0.5155	0.5246	0.6517	0.4033	
NLMO	0.3386	0.5159	0.5966	0.2972	
	Rh	Rh-Cl	Rh-P3	Rh-P1	Rh-P2
total	4.1082	0.3808	0.511	0.4498	0.442
covalent		0.1837	0.2918	0.246	0.239
ionic		0.1971	0.2192	0.2038	0.203

	Wilkinson				
	Rh-P2	Rh-P1	Rh-P3	Rh-Cl	
MBO	0.9554	0.8307	0.9865	0.9043	
WBI	0.4634	0.5091	0.6087	0.3551	
NLMO	0.3598	0.5508	0.6017	0.2546	
	Rh	Rh-Cl	Rh-P3	Rh-P1	Rh-P2
total	4.1458	0.3244	0.5298	0.4721	0.3821
covalent		0.1352	0.3094	0.2815	0.2102
ionic		0.1893	0.2204	0.1906	0.1719
	Planar				
	Rh-P2	Rh-P1	Rh-P3	Rh-Cl	
MBO	0.8862	0.7914	1.0238	0.9124	
WBI	0.4515	0.4980	0.6088	0.3637	
NLMO	0.3536	0.5392	0.6975	0.2710	
	Rh	Rh-Cl	Rh-P3	Rh-P1	Rh-P2
total	4.1515	0.3325	0.5160	0.4395	0.4090
covalent		0.1482	0.3077	0.2599	0.2311
ionic		0.1843	0.2084	0.1796	0.1779
	Dim				
	Rh1-P20	Rh1-P23	Rh1-Cl21	Rh1-Cl22	Rh2-P91
MBO	1.0464	1.1538	0.4666	0.448	1.0462
WBI	0.6744	0.6483	0.296	0.3159	0.6745
NLMO	0.6466	0.6278	0.2101	0.2247	0.6461
	Rh1-P20	Rh1-P23	Rh1-Cl21	Rh1-Cl22	Rh1
total	0.5729	0.5804	0.1316	0.2144	4.3454
covalent	0.3817	0.3844	0.0366	0.0536	
ionic	0.1912	0.196	0.095	0.1608	

Table SI11: Bond parameters of systems calculated at the X2C-r2SCAN/[x2c-def2-TZVPall (non-Rh) + x2c-def2-TZVPPall (Rh)] level of theory

	DSPC							
	Rh-P2	Rh-P1	Rh-P3	Rh-Cl				
MBO	0.7992	0.8653	1.0901	0.8087				
WBI	0.505	0.551	0.6587	0.4042				
NLMO	0.3286	0.5492	0.5888	0.2898				
	Rh	Rh-Cl	Rh-P3	Rh-P1	Rh-P2			
t	4.1046	0.3772	0.5181	0.4683	0.4271			
c		0.173	0.2869	0.2694	0.2258			
i		0.2042	0.2312	0.1989	0.2013			
	SPC							
	Rh-P2	Rh-P1	Rh-P3	Rh-Cl				
MBO	0.8109	0.8189	1.1076	0.7593				
WBI	0.5181	0.5274	0.6613	0.3966				

NLMO	0.3423	0.5278	0.6145	0.2957				
	Rh	Rh-Cl	Rh-P3	Rh-P1	Rh-P2			
t	4.1135	0.3699	0.5166	0.4473	0.4392			
c		0.1796	0.3031	0.2507	0.2433			
i		0.1904	0.2135	0.1966	0.1959			
	Wilkinson							
	Rh-P2	Rh-P1	Rh-P3	Rh-Cl				
MBO	0.8462	0.9768	1.0801	0.977				
WBI	0.5106	0.4645	0.6179	0.3463				
NLMO	0.3462	0.5643	0.6186	0.255				
	Rh	Rh-Cl	Rh-P3	Rh-P1	Rh-P2			
t	4.1544	0.3132	0.5324	0.4673	0.3784			
c		0.1304	0.3205	0.2871	0.2144			
i		0.1828	0.2119	0.1802	0.1639			
	Planar							
	Rh-P2	Rh-P1	Rh-P3	Rh-Cl				
MBO	0.8281	0.8591	1.1081	0.9946				
WBI	0.4518	0.4999	0.6189	0.3550				
NLMO	0.3533	0.5536	0.6281	0.2675				
	Rh	Rh-Cl	Rh-P3	Rh-P1	Rh-P2			
t	4.1516	0.3244	0.5249	0.4670	0.3814			
c		0.1442	0.3209	0.2843	0.2218			
i		0.1802	0.2032	0.1827	0.1597			
	Dim							
	Rh1-P20	Rh1-P23	Rh1-Cl21	Rh1-Cl22	Rh2-P91	Rh2-P92	Rh2-Cl21	Rh2-Cl22
MBO	1.099	1.1982	0.484	0.4664	1.0994	1.1989	0.4659	0.484
WBI	0.6835	0.6568	0.2881	0.3089	0.6835	0.6567	0.3089	0.2881
NLMO	0.6639	0.6453	0.2066	0.2204	0.664	0.6453	0.2204	0.2065
	Rh1-P20	Rh1-P23	Rh1-Cl21	Rh1-Cl22	Rh1			
t	0.5729	0.5804	0.1316	0.2144	4.3454			
c	0.3817	0.3844	0.0366	0.0536				
i	0.1912	0.196	0.095	0.1608				

Table SI12: Bond parameters of systems calculated at the X2C-CCSD/[x2c-def2-SVP (non-Rh) + x2c-def2-TZVPPall (Rh)] level of theory

	DSPC				
	Rh-P2	Rh-P1	Rh-P3	Rh-Cl	
MBO	0.6958	0.6286	0.8015	0.9959	
WBI	0.4747	0.4294	0.5414	0.4078	
NLMO	0.4594	0.2949	0.4851	0.2874	
Natural Bonds					
	Rh	Rh-Cl	Rh-P3	Rh-P1	Rh-P2
t	4.1167	0.392	0.4913	0.4627	0.4206
c	-	0.1527	0.2313	0.2264	0.1872

i	-	0.2393	0.26	0.2363	0.2334

	SPC			
	Rh-P2	Rh-P1	Rh-P3	Rh-Cl
MBO	0.6705	0.6886	0.8685	0.9556
WBI	0.4456	0.4547	0.5507	0.4
NLMO	0.349	0.4502	0.5204	0.2839

	Rh	Rh-Cl	Rh-P3	Rh-P1	Rh-P2
t	4.1248	0.3831	0.4921	0.4417	0.4335
c	-	0.1622	0.2511	0.2127	0.2059
i	-	0.2209	0.241	0.229	0.2276

QTAIM

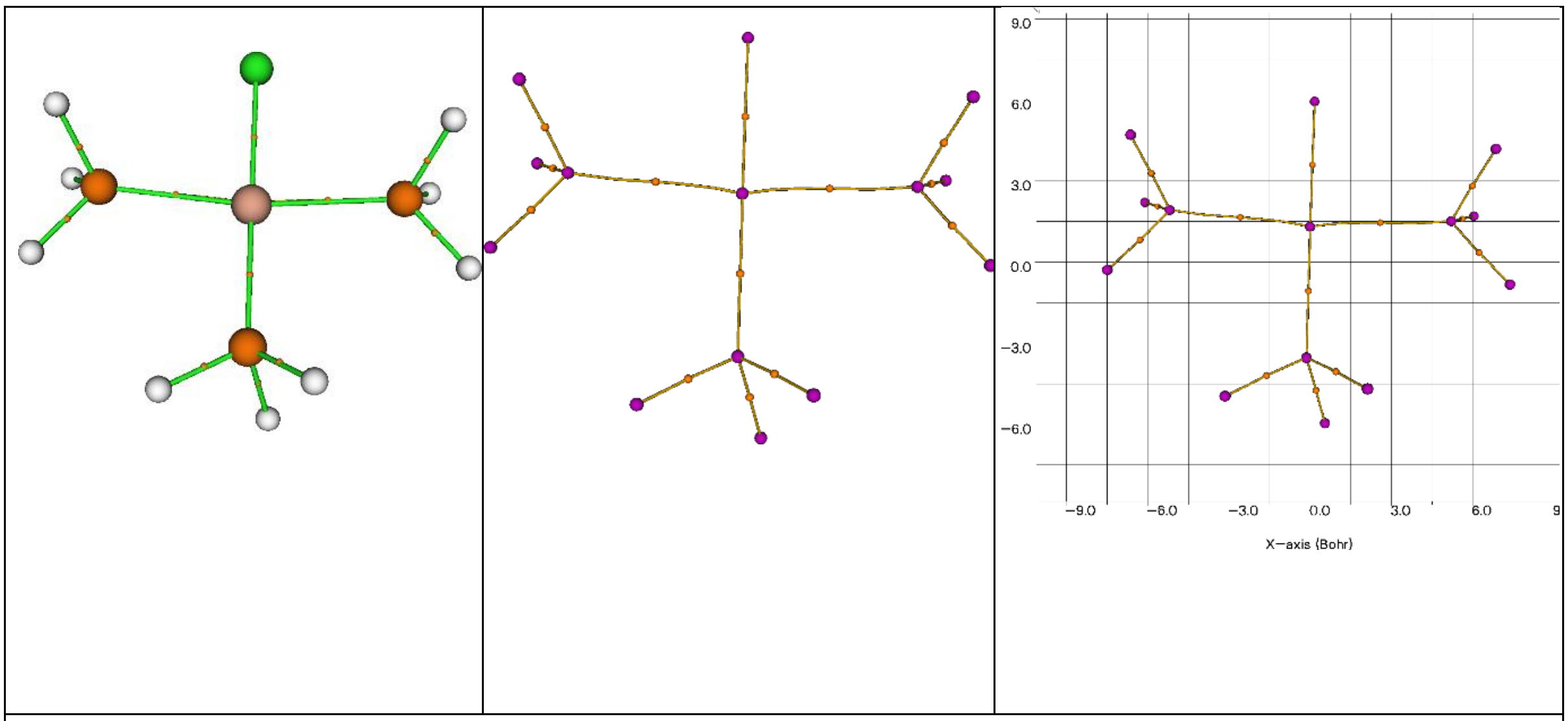

SPC

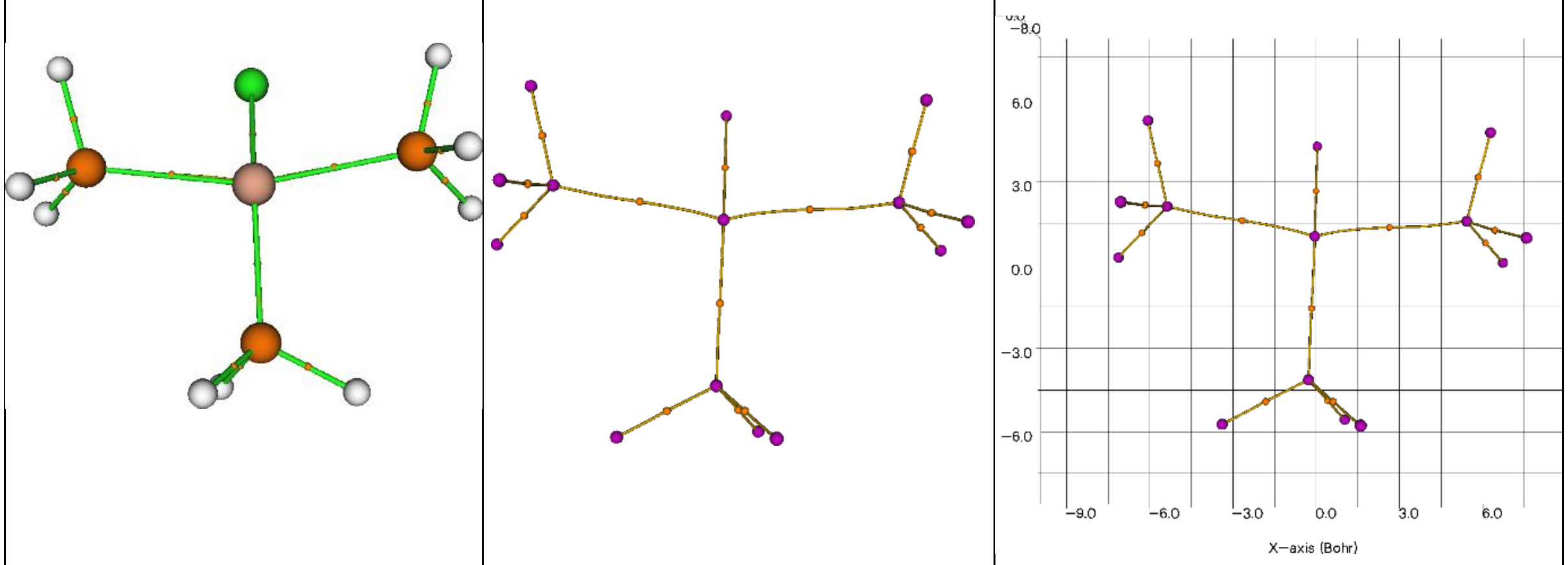

DSPC

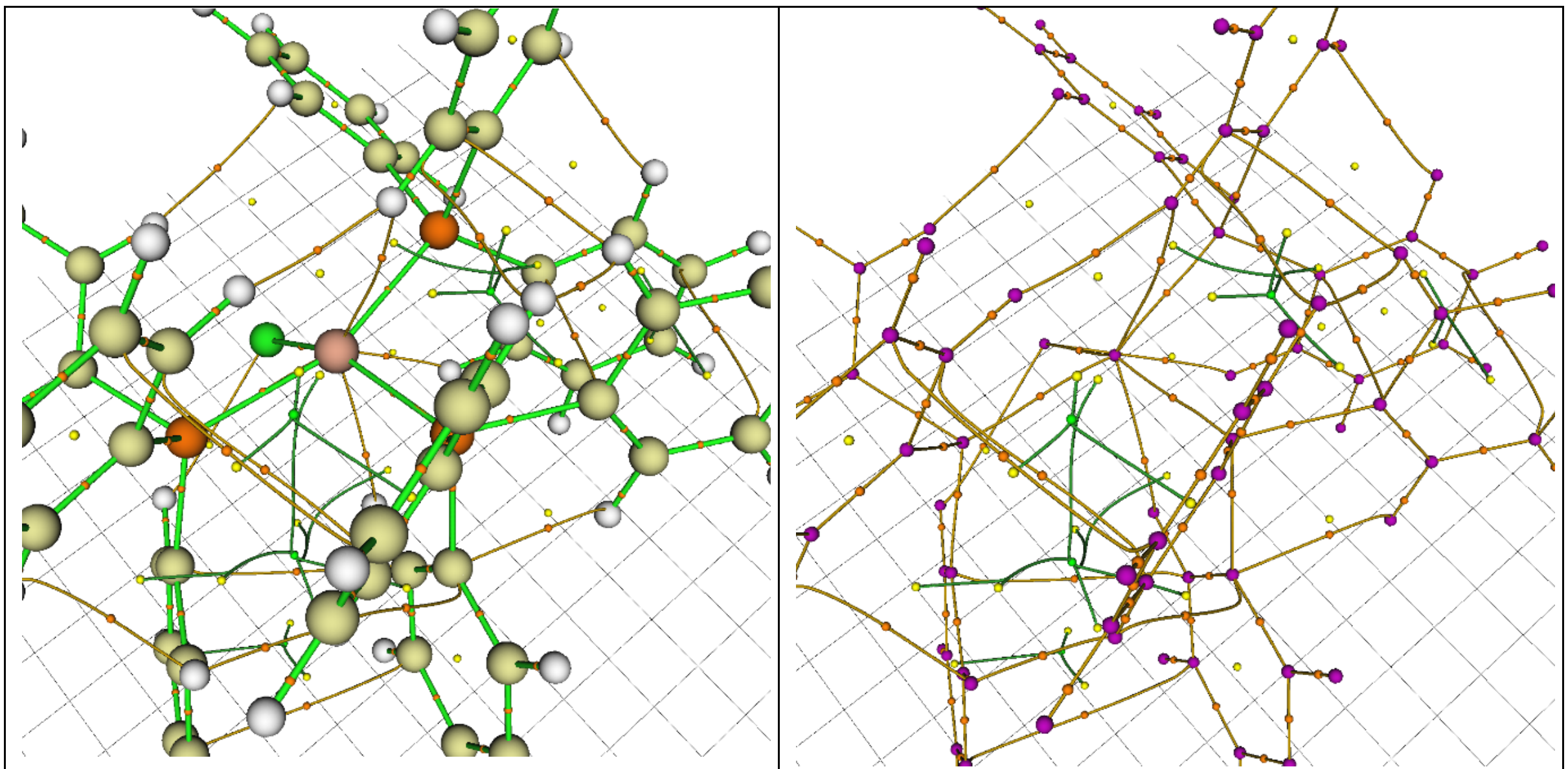

Wilkinson

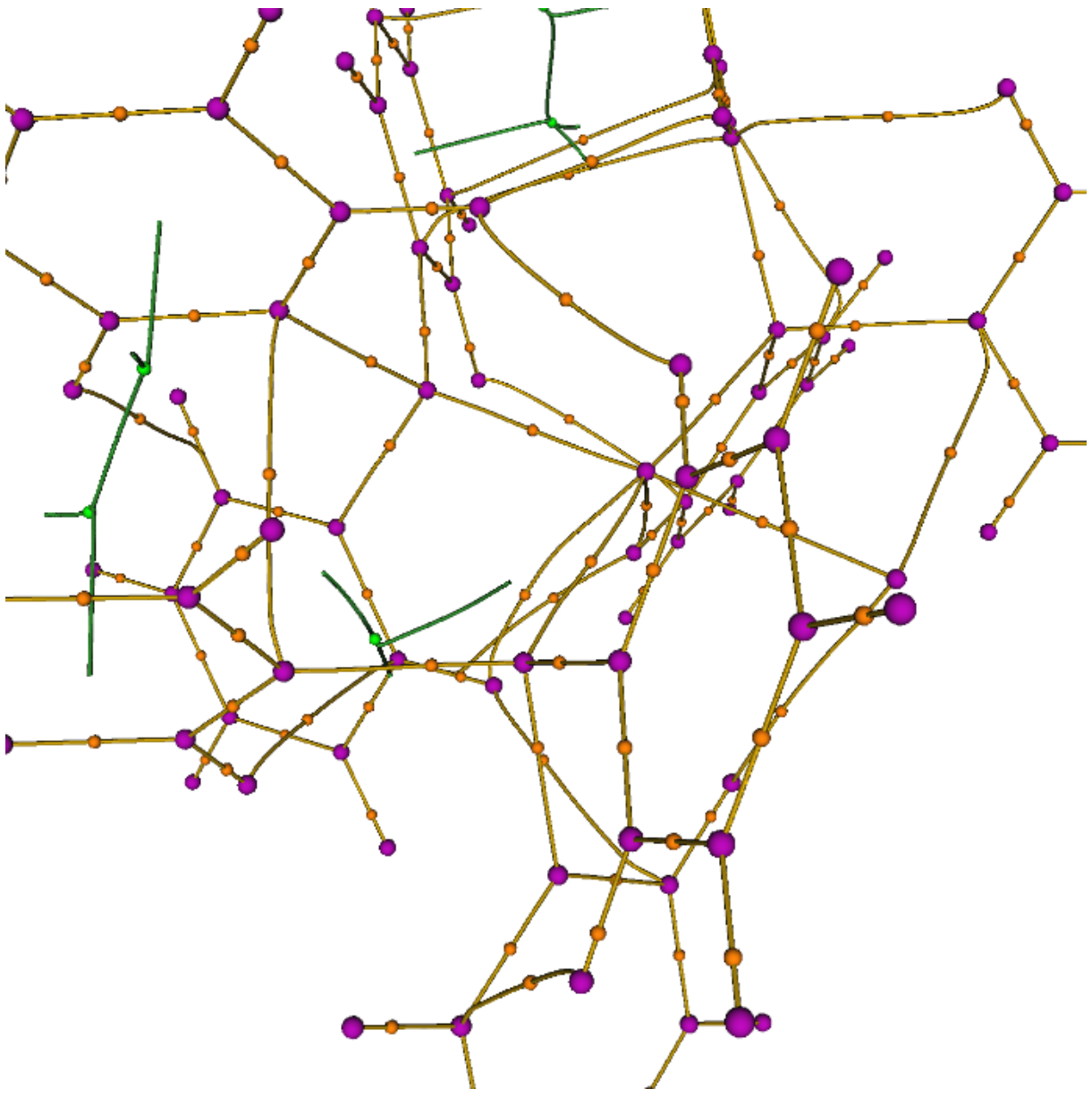

Wilkinson (planar)

Figure SI2: Graphic representation of critical points of monomers at the X2C-TPSSh/[x2c-def2-TZVPall (non-Rh) + x2c-def2-TZVPPall (Rh)] level of theory

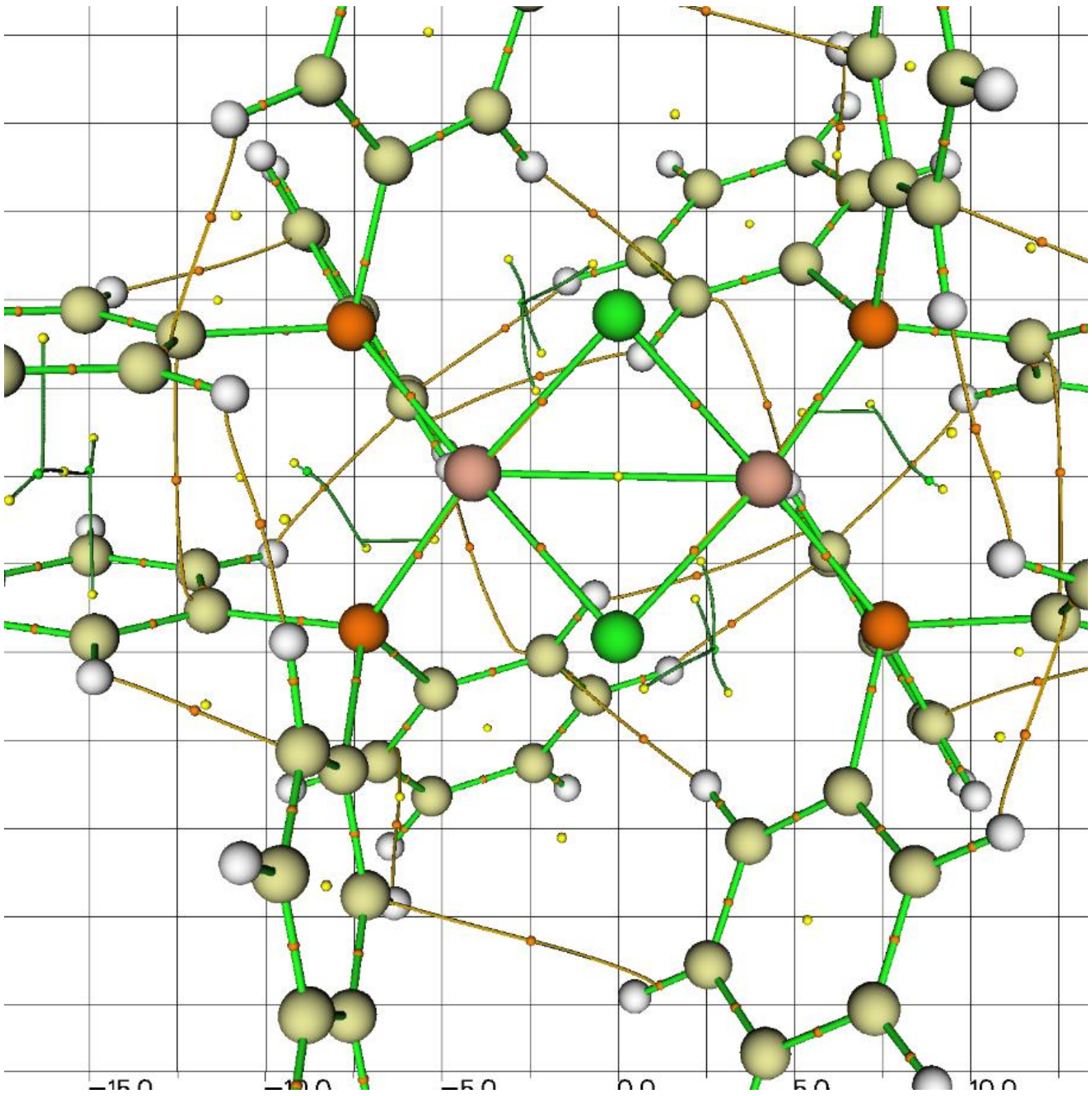

Figure SI3: Graphic representation of critical points of dimer at the X2C-TPSSh/[x2c-def2-TZVPall (non-Rh) + x2c-def2-TZVPPall (Rh)] level of theory

CCSD

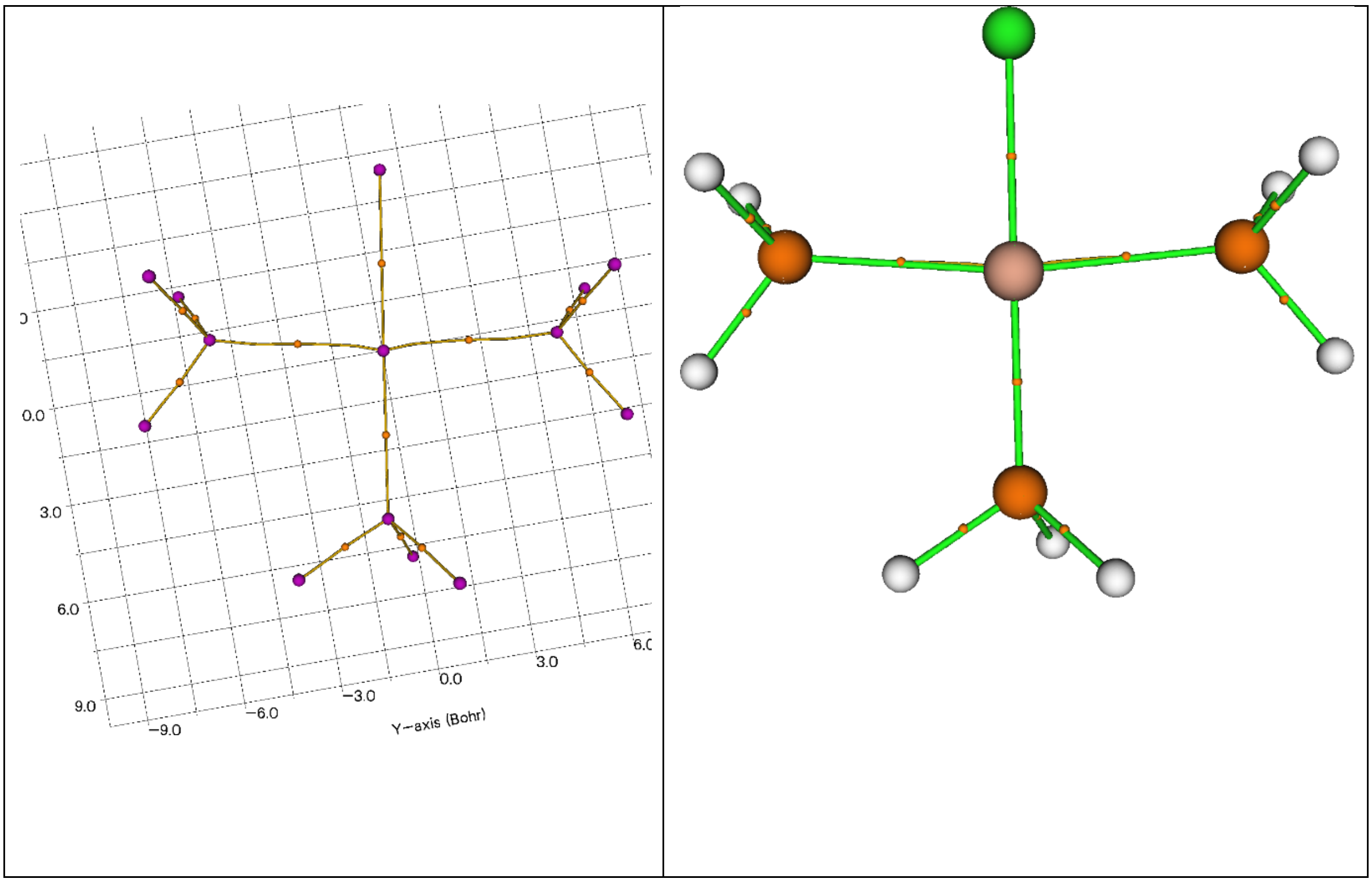

DSPC

Figure SI4: Graphic representation of critical points of reduced model systems at X2C-CCSD/[x2c-def2-SVP (non-Rh) + x2c-def2-TZVPPall (Rh)] level of theory

NPA CHARGES

Table SI13: NPA charges (in e) of systems calculated at the X2C-TPSSh/[x2c-def2-TZVPall (non-Rh) + x2c-def2-TZVPPall (Rh)] level of theory

	SPC	DSPC	Full	Planar
Rh	-0.43186	-0.38584	-0.38192	-0.45381
P2	0.292	0.26088	1.09527	1.10893
P1	0.30032	0.3	1.13633	1.13179
P3	0.32267	0.28572	1.13444	1.16022
Cl	-0.53716	-0.56279	-0.55944	-0.53221

	Dimer
Rh	-0.33928
P2	1.19532
P1	1.17887
Cl2	-0.37333
Cl1	-0.37333

Table SI14: NPA (in e) charges of systems calculated at the X2C-r2SCAN/[x2c-def2-TZVPall (non-Rh) + x2c-def2-TZVPPall (Rh)] level of theory

	SPC	DSPC	Full	Planar
Rh	-0.44332	-0.3961	-0.38576	-0.45998
P2	0.24881	0.21599	1.09847	1.11125
P1	0.25774	0.25755	1.14149	1.13594
P3	0.28244	0.24624	1.13959	1.16375
Cl	-0.53636	-0.56093	-0.5595	-0.53294

	Dimer
Rh	-0.34125
P2	1.20211
P1	1.18447
Cl2	-0.37737
Cl1	-0.37735

Table SI15: NPA charges of systems calculated at the X2C-CCSD/[x2c-def2-SVP (non-Rh) + x2c-def2-TZVPPall (Rh)] level of theory

	SPC	DSPC
Rh	-0.43536	-0.38864
P2	0.29081	0.25849
P1	0.30011	0.29838
P3	0.32522	0.28618
Cl	-0.52826	-0.5512

Table SI16: X2C-TPSSh/[x2c-def2-TZVPall (non-Rh) + x2c-def2-TZVPPall (Rh)] bond force constants

	Direction	Ha/A**2
SPC	P-Rh-P	0.37172912
	P-Rh-Cl	0.508115968
Wilkinson	P-Rh-P	0.385697857
	P-Rh-Cl	0.521195136
Dimer	P-Rh-P	1.031411649

Table SI17: Bond orders for [Rh(PPh3)2Cl] fragments (X2C-TPSSh/[x2c-def2-TZVPall (non-Rh) + x2c-def2-TZVPPall (Rh)])

	Cis			
	Rh-P1	Rh-P3	Rh-Cl	Total
MBO	1.2269	1.0344	0.8937	3.155
WBI	0.8496	0.6678	0.427	1.9444
NLMO	0.7887	0.6866	0.2744	1.7497
	Trans			
	Rh-P1	Rh-P2	Rh-Cl	Total
MBO	0.8243	0.8525	0.939	2.6158
WBI	0.497	0.5077	0.5239	1.5286
NLMO	0.3828	0.5153	0.4493	1.3474

Table SI18: Bond orders for [Rh(PPh3)2Cl] fragments (X2C-r2SCAN/[x2c-def2-TZVPall (non-Rh) + x2c-def2-TZVPPall (Rh)])

	Cis			
	Rh-P1	Rh-P3	Rh-Cl	Total
MBO	1.2922	1.101	0.9268	3.32
WBI	0.8542	0.6755	0.4207	1.9504
NLMO	0.8134	0.7034	0.2715	1.7883
	Trans			
	Rh-P1	Rh-P2	Rh-Cl	Total
MBO	0.8495	0.8866	1.0068	2.7429
WBI	0.4953	0.5092	0.5177	1.5222
NLMO	0.388	0.5315	0.453	1.3725

Table SI19: Bond orders for [H2Rh(PPh3)2Cl] fragments (X2C-TPSSh/[x2c-def2-TZVPall (non-Rh) + x2c-def2-TZVPPall (Rh)])

	Trans					
	Rh-H1	R-H2	Rh-P1	Rh-P2	Rh-Cl	Total
MBO	0.872	0.873	0.8178	0.8161	0.7216	4.1005
WBI	0.6223	0.6216	0.4585	0.4584	0.3239	2.4847
NLMO	0.7451	0.7474	0.4063	0.5711	0.1738	2.6437
	Cis					
	Rh-H1	R-H2	Rh-P1	Rh-P3	Rh-Cl	Total
MBO	0.8917	0.7967	0.6664	1.0173	0.8715	4.2436
WBI	0.7236	0.5236	0.2994	0.5851	0.389	2.5207
NLMO	0.8094	0.7137	0.2028	0.6673	0.2813	2.6745
	Syn					
	Rh-H1	R-H2	Rh-P1	Rh-P2	Rh-Cl	Total
MBO	0.8071	0.8138	1.2014	0.6705	0.7361	4.2289
WBI	0.5364	0.5731	0.7552	0.2909	0.2958	2.4514
NLMO	0.6924	0.893	0.8355	0.1924	0.178	2.7913

Table SI20: Bond orders for [H2Rh(PPh3)2Cl] fragments (X2C-r2SCAN/[x2c-def2-TZVPall (non-Rh) + x2c-def2-TZVPPall (Rh)])

	Trans					
	Rh-H1	R-H2	Rh-P1	Rh-P2	Rh-Cl	Total
MBO	0.884	0.8842	0.8293	0.8282	0.77	4.1957
WBI	0.6136	0.6128	0.4592	0.4591	0.3155	2.4602
NLMO	0.7295	0.7307	0.4103	0.5865	0.1689	2.6259
	Cis					
	Rh-H1	R-H2	Rh-P1	Rh-P3	Rh-Cl	Total
MBO	0.8993	0.7976	0.697	1.0686	0.8804	4.3429
WBI	0.7107	0.5112	0.3017	0.5928	0.3808	2.4972
NLMO	0.7944	0.6909	0.2037	0.6863	0.2773	2.6526
	Syn					
	Rh-H1	R-H2	Rh-P1	Rh-P2	Rh-Cl	Total
MBO	0.8052	0.8308	1.2427	0.7143	0.7424	4.3354
WBI	0.5235	0.5658	0.7571	0.2938	0.2888	2.429
NLMO	0.6914	0.7856	0.8541	0.1918	0.1738	2.6967

Table SI21: [H2Rh(PPh3)2Cl] structures

a) trans

```
Rh   0.0376702   1.3267366  -0.0098590
C    3.1346828   0.1631939   1.1899088
C    2.6353592   0.1826544   2.5031827
C    3.2468286  -0.5791035   3.5012012
C    4.3546017  -1.3785956   3.1943880
C    4.8500815  -1.4092215   1.8871528
C    4.2461734  -0.6389301   0.8874916
C    3.1421419   2.8541002   0.1627944
C    4.2335520   3.0371427   1.0258243
C    4.8124614   4.3037175   1.1621680
```

C	4.3083249	5.3885084	0.4377331
C	3.2191931	5.2074862	-0.4238903
C	2.6317520	3.9478195	-0.5595744
C	3.0146804	0.6098938	-1.6530479
C	2.3355228	-0.4434825	-2.2865266
C	2.8250948	-0.9741698	-3.4812313
C	3.9878536	-0.4473735	-4.0568682
C	4.6603297	0.6093761	-3.4342619
C	4.1768776	1.1383739	-2.2328830
P	2.3339543	1.2255587	-0.0724004
Cl	-0.0678969	2.2699209	-2.2200231
P	-2.2497876	1.3853318	0.2200891
C	-2.8917405	1.1691343	1.9243286
C	-3.9562085	1.9373323	2.4208359
C	-4.4350755	1.7200241	3.7174358
C	-3.8613814	0.7312824	4.5226067
C	-2.8002927	-0.0388998	4.0309844
C	-2.3129610	0.1832392	2.7414029
C	-3.1358637	0.0976579	-0.7373128
C	-2.7401706	-0.1153860	-2.0700753
C	-3.3952007	-1.0738828	-2.8463776
C	-4.4379634	-1.8318796	-2.2992583
C	-4.8277238	-1.6265295	-0.9719472
C	-4.1807660	-0.6630949	-0.1902937
C	-3.0048374	2.9554875	-0.3330318
C	-2.3059299	4.1432430	-0.0628537
C	-2.8474833	5.3746935	-0.4356437
C	-4.0834336	5.4262266	-1.0918146
C	-4.7768253	4.2442044	-1.3724158
C	-4.2407297	3.0090104	-0.9933654
H	1.7601077	0.7926941	2.7373164
H	2.8527949	-0.5551101	4.5199493
H	4.8274964	-1.9812277	3.9735018
H	5.7110224	-2.0353035	1.6412096
H	4.6396694	-0.6649997	-0.1305415
H	4.6322748	2.1934076	1.5926008
H	5.6607440	4.4409512	1.8370792
H	4.7620560	6.3766030	0.5465535
H	2.8211767	6.0530906	-0.9899124
H	1.7765497	3.7992326	-1.2253746
H	1.4156132	-0.8346127	-1.8439990
H	2.2923388	-1.7922257	-3.9715410
H	4.3655874	-0.8566271	-4.9969448
H	5.5633187	1.0267991	-3.8860559
H	4.7008074	1.9661520	-1.7499723
H	-4.4105531	2.7088543	1.7961688
H	-5.2597904	2.3273086	4.0978845
H	-4.2366040	0.5634646	5.5349521
H	-2.3454032	-0.8097709	4.6575212
H	-1.4732500	-0.4044788	2.3636227
H	-1.9208211	0.4770214	-2.4877630
H	-3.0864975	-1.2331970	-3.8823034
H	-4.9447409	-2.5851974	-2.9073320
H	-5.6393933	-2.2172518	-0.5404878
H	-4.4902916	-0.5055661	0.8448349
H	-1.3307750	4.0941725	0.4288922
H	-2.2993028	6.2959143	-0.2251612
H	-4.5027543	6.3895791	-1.3918914
H	-5.7376116	4.2820450	-1.8912718
H	-4.7815130	2.0867283	-1.2168219

H	0.0527850	0.0467926	0.8381032
H	0.1367408	1.6063522	1.4966496

b) cis

Rh	0.0174157	0.2800991	-0.5778678
C	-1.7534111	-2.5377704	0.3745589
C	-2.8155723	-1.6930785	0.7263670
C	-4.0691003	-2.2310455	1.0372343
C	-4.2716310	-3.6133392	0.9864747
C	-3.2153055	-4.4604077	0.6296432
C	-1.9596872	-3.9267821	0.3317916
C	0.6074042	-2.7972675	-1.3433590
C	1.1561399	-4.0853459	-1.2281682
C	1.6875352	-4.7245066	-2.3516882
C	1.6780563	-4.0852271	-3.5958548
C	1.1288424	-2.8053850	-3.7194970
C	0.5946179	-2.1663949	-2.5995907
C	0.8238785	-2.4571249	1.5343044
C	0.1671472	-2.5233458	2.7737609
C	0.8604714	-2.9293402	3.9170610
C	2.2128484	-3.2766235	3.8340809
C	2.8746153	-3.2057973	2.6046141
C	2.1856351	-2.7902959	1.4641458
P	-0.0813774	-1.8407301	0.0622984
C	3.1374981	0.2841085	1.3967834
C	2.3247829	0.2425171	2.5402805
C	2.8864525	0.0085521	3.7967252
C	4.2638591	-0.2004878	3.9176091
C	5.0804953	-0.1558046	2.7818411
C	4.5232288	0.0998538	1.5260463
C	2.8389353	2.5120322	-0.2764656
C	4.0753191	2.9011710	-0.8121905
C	4.4560355	4.2463875	-0.7889672
C	3.6083023	5.2056600	-0.2243508
C	2.3760879	4.8186020	0.3147695
C	1.9886522	3.4770917	0.2841767
C	3.2235074	-0.0542168	-1.5490738
C	3.9439485	-1.2510268	-1.4167363
C	4.4609883	-1.8937703	-2.5455619
C	4.2705043	-1.3443386	-3.8161540
C	3.5505406	-0.1522738	-3.9564262
C	3.0204204	0.4857898	-2.8340766
P	2.3254236	0.7538778	-0.1747387
Cl	-0.0778274	2.1798439	-1.9888203
H	1.2475556	0.3896861	2.4345924
H	2.2458007	-0.0232161	4.6802321
H	4.7039071	-0.3949273	4.8986475
H	6.1587136	-0.3063337	2.8760566
H	5.1720118	0.1666237	0.6499937
H	4.7398937	2.1563487	-1.2560419
H	5.4176018	4.5453681	-1.2132893
H	3.9060690	6.2569073	-0.2089401
H	1.7078876	5.5662453	0.7484100
H	1.0186461	3.1742149	0.6835230
H	4.1154452	-1.6820519	-0.4301756
H	5.0157381	-2.8273212	-2.4279113
H	4.6761848	-1.8474369	-4.6969999
H	3.3907943	0.2794527	-4.9472711
H	2.4257150	1.3961771	-2.9470774
H	3.9332093	-3.4639000	2.5337164

H	0.3389294	-2.9798893	4.8757329
H	2.7519126	-3.5970686	4.7285166
H	2.7067894	-2.7333973	0.5092735
H	-2.6576590	-0.6128905	0.7405600
H	-4.8905318	-1.5648710	1.3107799
H	-5.2532919	-4.0323529	1.2199590
H	-3.3700209	-5.5408653	0.5822103
H	-1.1426344	-4.5971590	0.0588231
H	1.1835721	-4.5887688	-0.2597610
H	2.1139812	-5.7255447	-2.2528382
H	2.1018591	-4.5851981	-4.4699000
H	1.1197438	-2.2992366	-4.6863732
H	0.1555273	-1.1679703	-2.7060670
H	-0.8917419	-2.2655749	2.8459001
H	-0.4716519	0.5609269	0.8354353
H	-1.5036386	0.0456000	-0.9581907

c) syn

Rh	0.0169389	0.3406101	-0.1365567
C	-1.8025037	-2.4567196	0.1551811
C	-2.6764058	-1.7139739	0.9696396
C	-3.9659826	-2.1863130	1.2215617
C	-4.4022807	-3.3864933	0.6477762
C	-3.5423732	-4.1151717	-0.1789972
C	-2.2435365	-3.6562175	-0.4238110
C	0.6050285	-2.8241318	-1.4460604
C	0.9263193	-4.1776978	-1.2229309
C	1.4325404	-4.9609038	-2.2617126
C	1.6200312	-4.4054289	-3.5325953
C	1.2938360	-3.0664644	-3.7610604
C	0.7885404	-2.2793272	-2.7231231
C	0.8107945	-2.4065244	1.3989071
C	0.2013819	-2.3574580	2.6628215
C	0.9170440	-2.7414312	3.7983431
C	2.2451617	-3.1684477	3.6866703
C	2.8610212	-3.2041173	2.4330038
C	2.1485900	-2.8208900	1.2953479
P	-0.0959381	-1.8215007	-0.0847431
C	3.3634621	0.3133229	1.5535864
C	2.6499714	0.1929398	2.7550251
C	3.3377707	0.0109929	3.9582770
C	4.7335128	-0.0606297	3.9672796
C	5.4489014	0.0747484	2.7704378
C	4.7685059	0.2746423	1.5672721
C	2.7323151	2.4411171	-0.2113073
C	3.8395390	2.9258887	-0.9261877
C	4.0560529	4.3026315	-1.0309573
C	3.1746547	5.2030900	-0.4205134
C	2.0764814	4.7235568	0.3010039
C	1.8529815	3.3473059	0.4066602
C	3.2832006	-0.1603212	-1.3438526
C	4.1051004	-1.2847300	-1.1772212
C	4.6451816	-1.9412741	-2.2888803
C	4.3875719	-1.4704043	-3.5779519
C	3.5774066	-0.3421350	-3.7535537
C	3.0186473	0.2986894	-2.6478219
P	2.3915248	0.6491847	0.0382073
Cl	-0.6592532	1.1604060	2.0416066
H	1.5584359	0.2596957	2.7406401
H	2.7768604	-0.0819106	4.8907994

H	5.2692165	-0.2118461	4.9077973
H	6.5410517	0.0364152	2.7768299
H	5.3339320	0.4060995	0.6418320
H	4.5319890	2.2301021	-1.4048645
H	4.9180344	4.6740929	-1.5905401
H	3.3460238	6.2787482	-0.5070015
H	1.3877561	5.4211968	0.7830827
H	1.0021332	2.9695270	0.9837869
H	4.3349674	-1.6500008	-0.1759065
H	5.2749598	-2.8216094	-2.1411725
H	4.8137916	-1.9809491	-4.4445414
H	3.3695122	0.0335261	-4.7582495
H	2.3668521	1.1631036	-2.7963298
H	3.9015942	-3.5222990	2.3393319
H	0.4337086	-2.7035148	4.7771554
H	2.8018050	-3.4639649	4.5787791
H	2.6296755	-2.8563106	0.3184382
H	-2.3431888	-0.7600631	1.3909388
H	-4.6369824	-1.6057176	1.8587542
H	-5.4161695	-3.7473655	0.8364444
H	-3.8819183	-5.0440961	-0.6431054
H	-1.5861887	-4.2303638	-1.0780887
H	0.7854471	-4.6205174	-0.2349823
H	1.6818329	-6.0083122	-2.0767591
H	2.0217138	-5.0181721	-4.3431065
H	1.4398275	-2.6258017	-4.7491427
H	0.5434931	-1.2307014	-2.8963438
H	-0.8280023	-2.0136361	2.7649367
H	-1.5091039	0.2655432	-0.5380517
H	0.2478400	0.1479632	-1.6602396

Table SI22: [Rh(PPh3)2Cl] resonance structures and NRT bonding characters

a) X2C-TPSSh/[x2c-def2-TZVPall (non-Rh) + x2c-def2-TZVPPall (Rh)]

Resonances	cis	trans
a	50.72	42.73
b	31.21	37.95
c	11.07	11.14
d	3.92	8.18
		Rh-Cl
tot	0.3121	0.8068
cov	0.1167	0.3615
Ion	0.1954	0.4453

b) X2C-r2SCAN/[x2c-def2-TZVPall (non-Rh) + x2c-def2-TZVPPall (Rh)]

Resonances	cis	trans
a	47.25	35.22
b	31.05	17.48
c	10.41	14.55
d	4.28	9.58
		Rh-Cl
t	0.3534	0.7203

c	0.1192	0.325
l	0.2342	0.3953

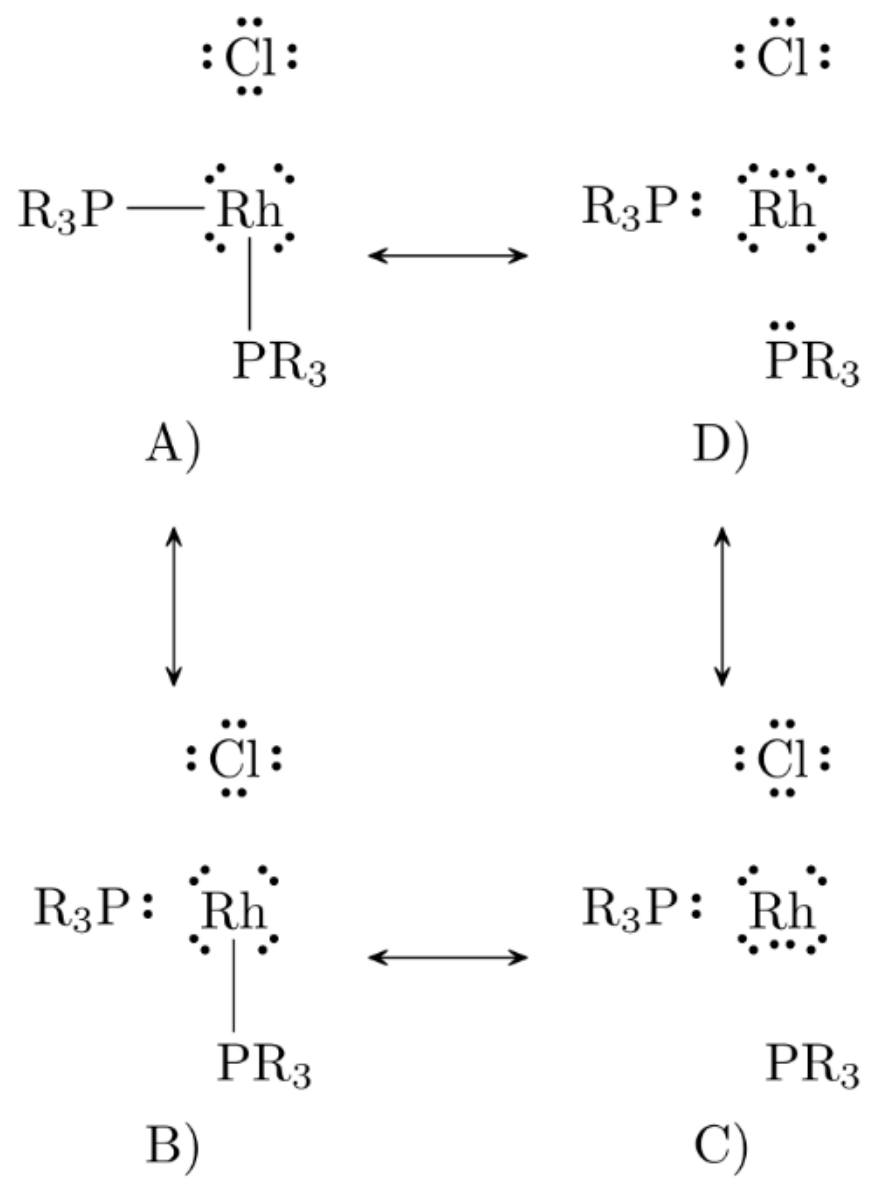

Figure SI5: cis fragment resonance structures

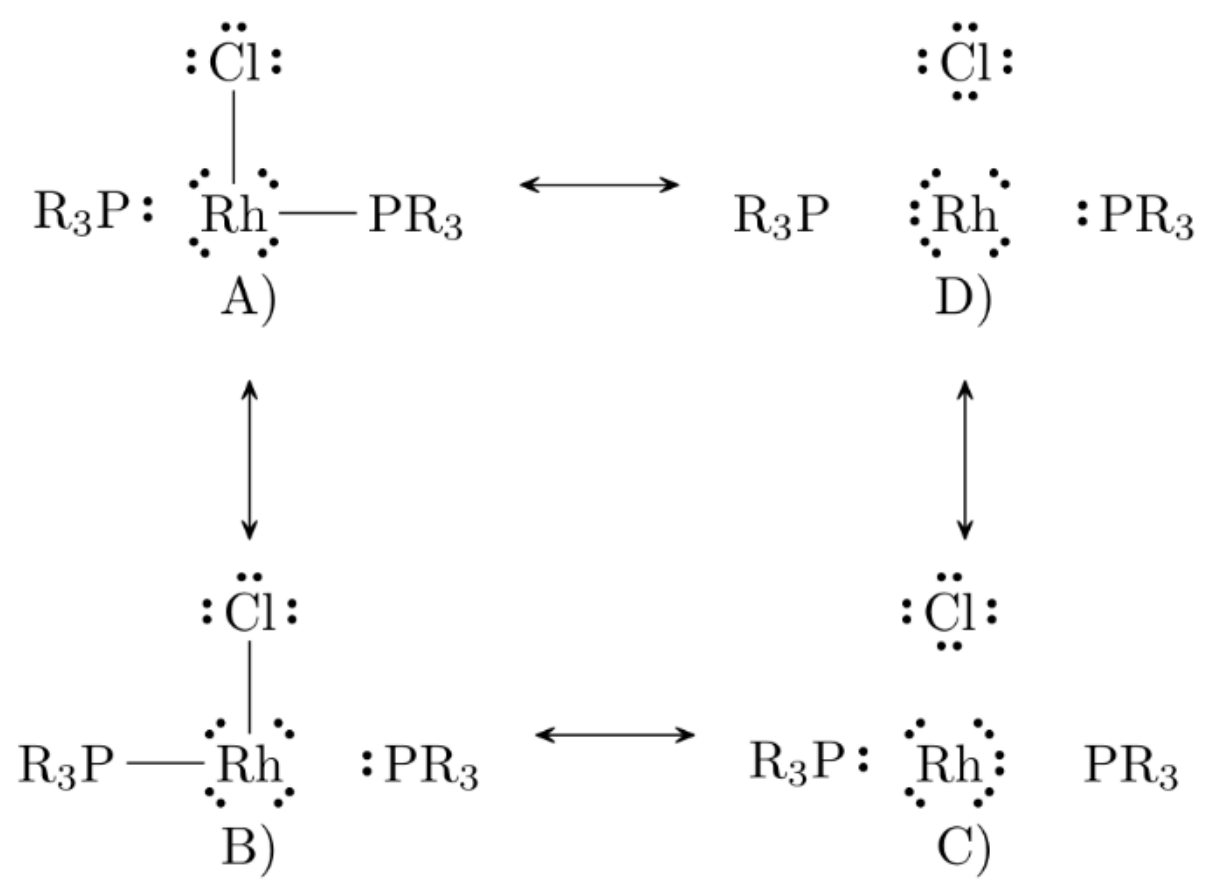

Figure SI6: trans fragment resonance structures

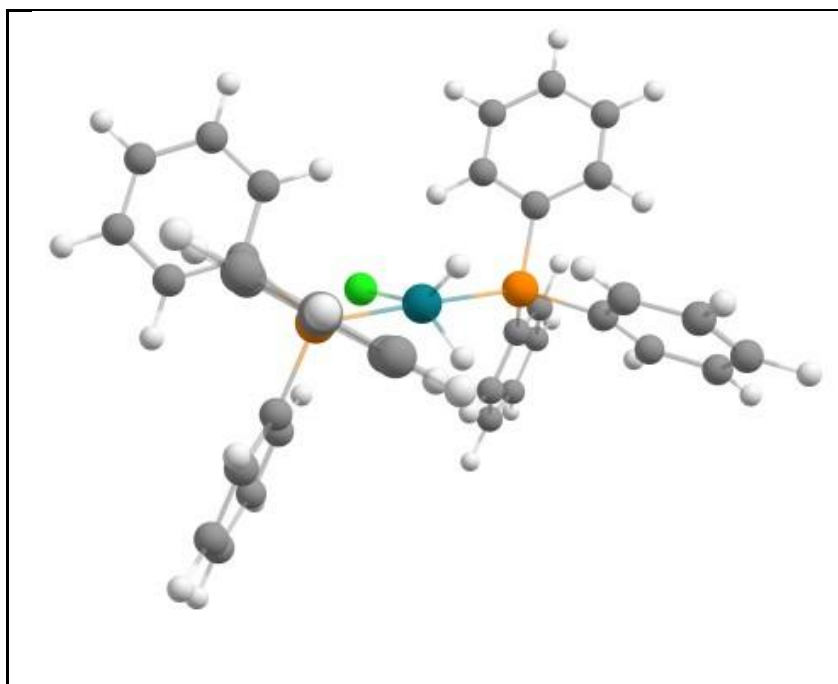	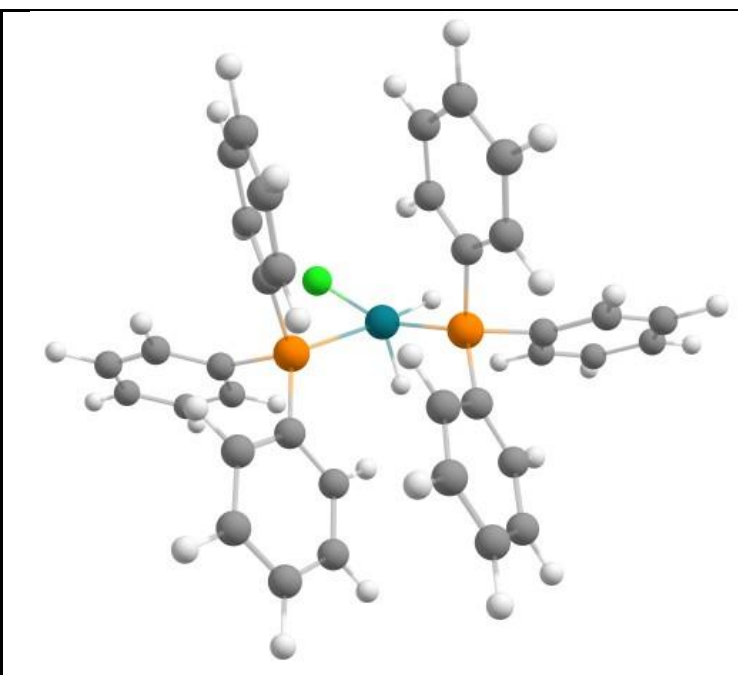	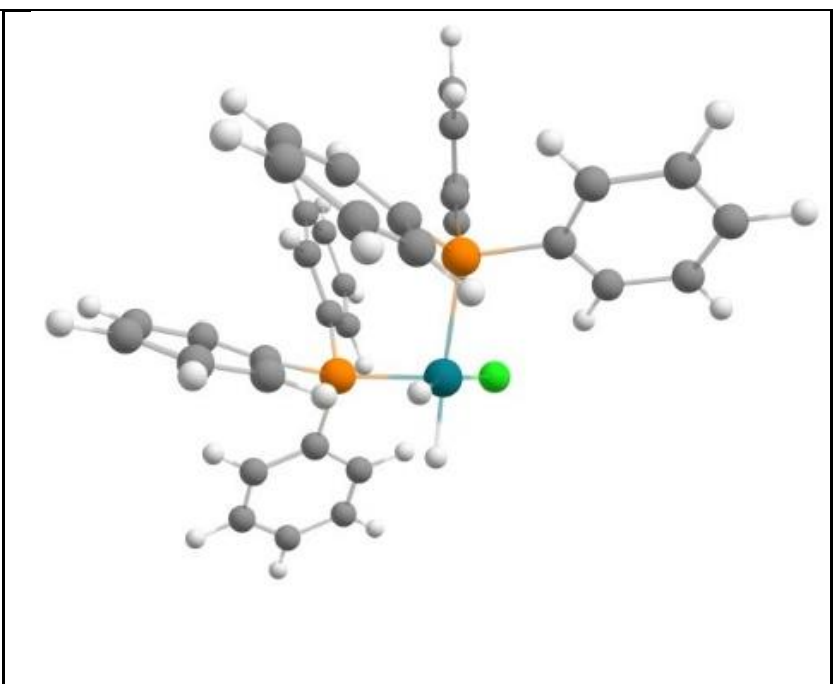
trans	cis	syn

Figure SI7: Structures of H2 coordinated on catalysator fragment

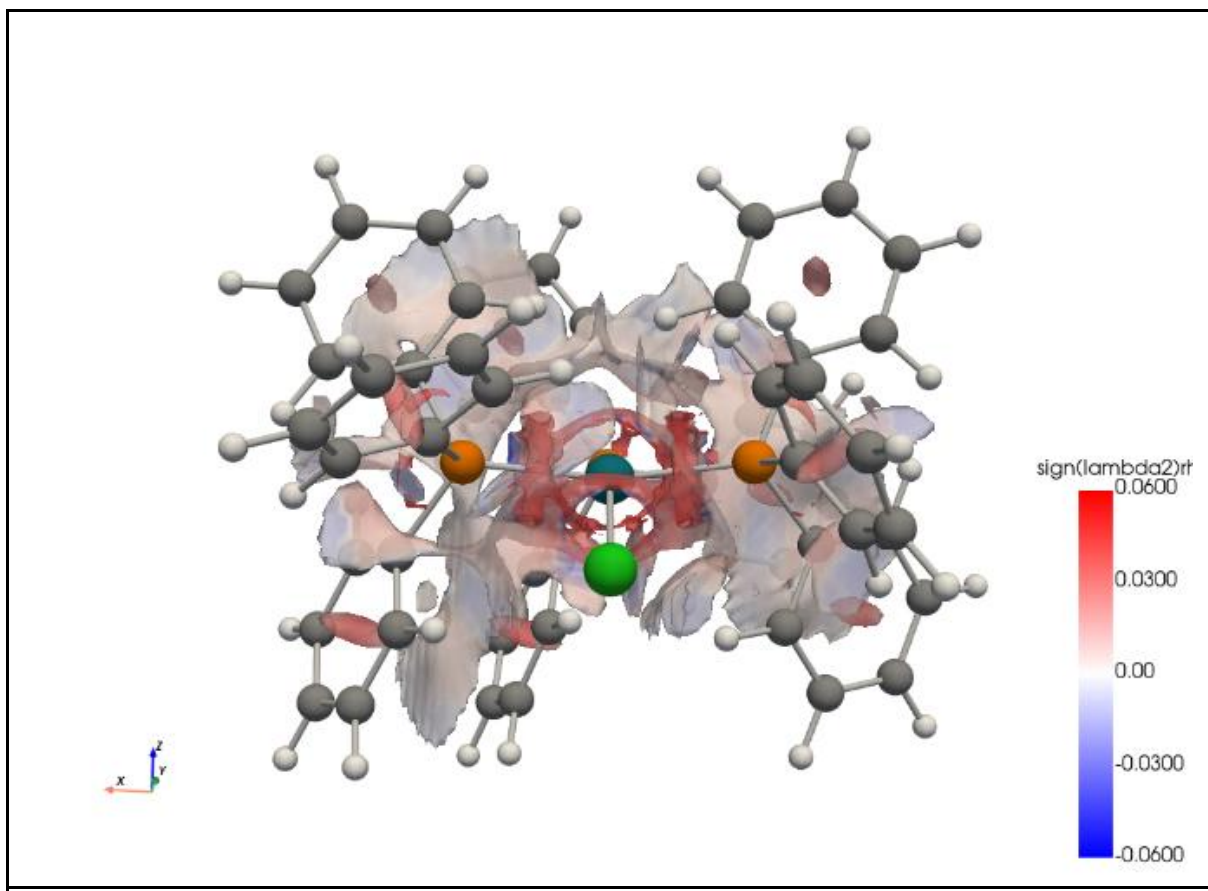

Wilkinson

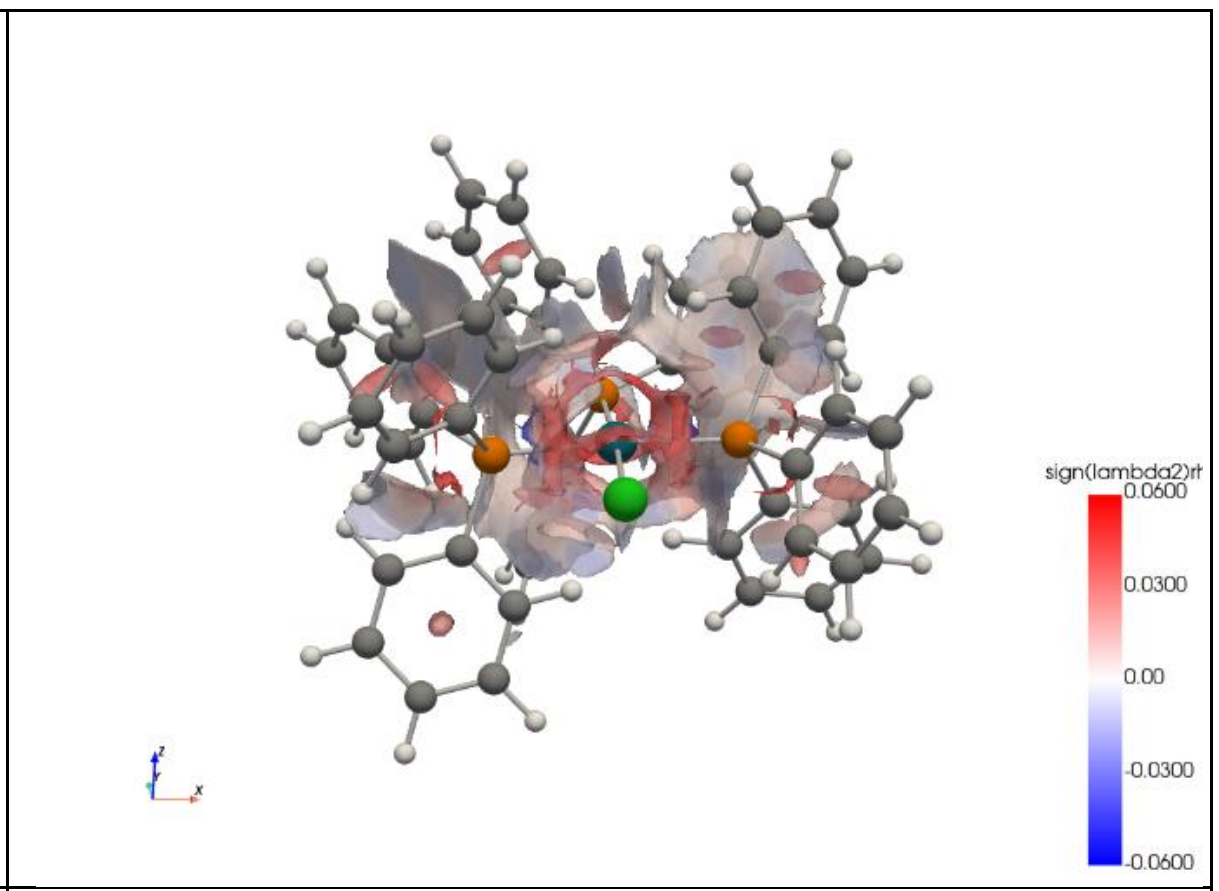

Wilkinson planar

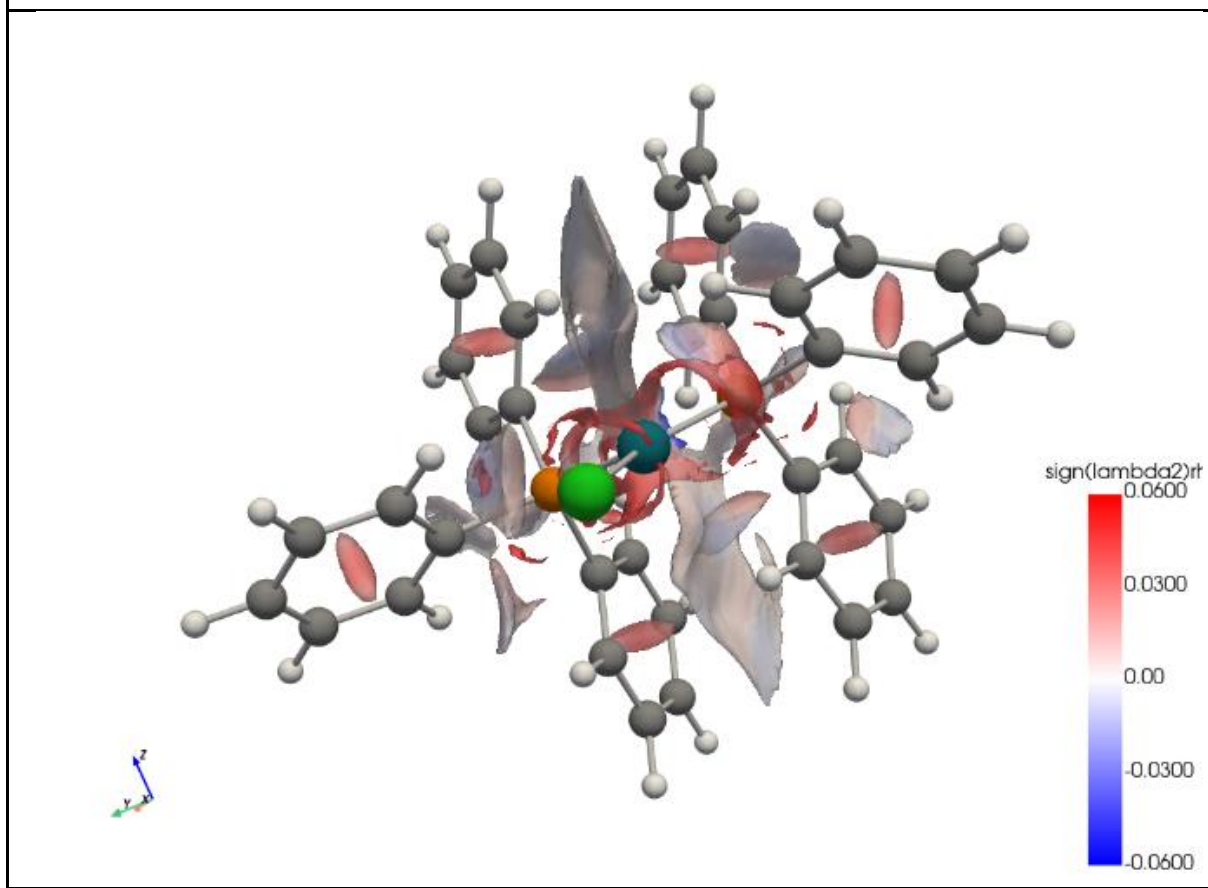

Cis fragment

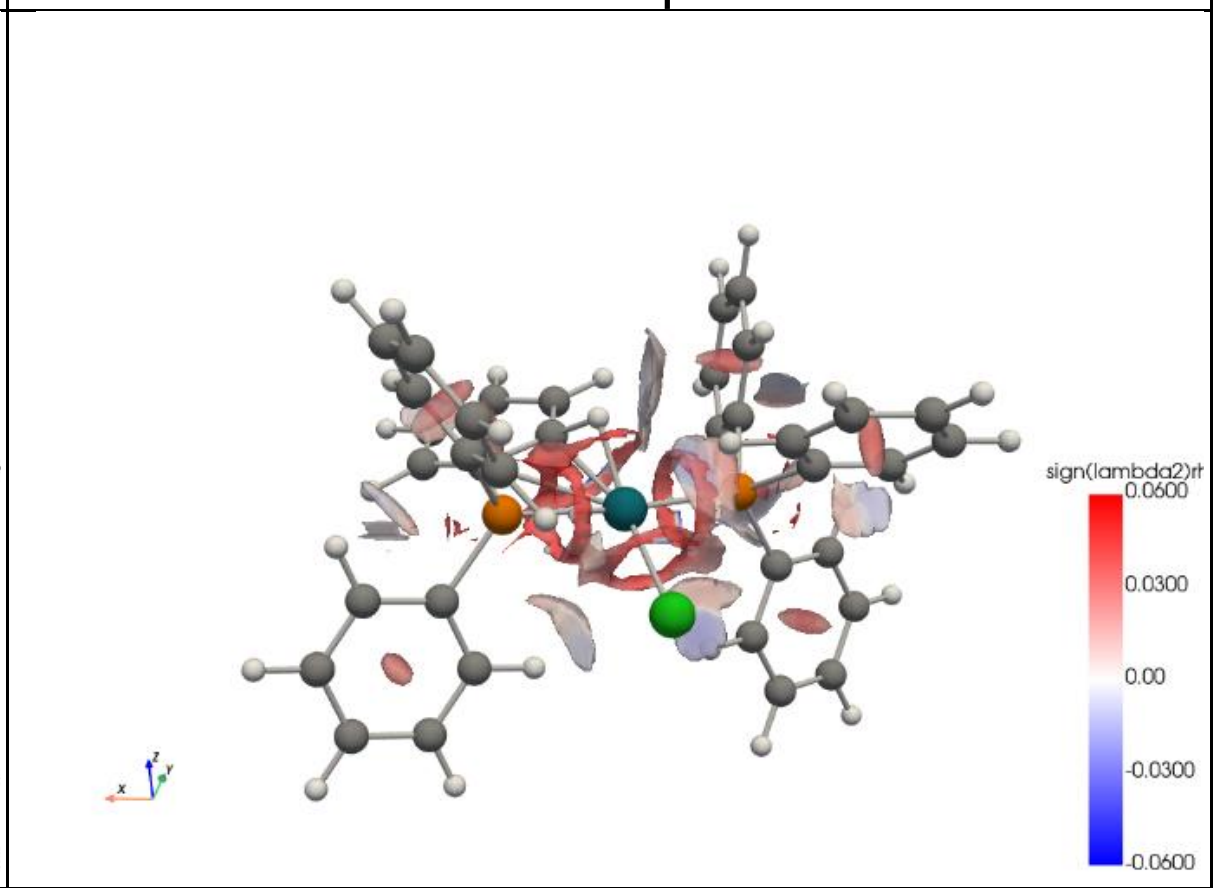

Trans fragment

Figure SI8: RDG/sign(lambda2 rho) plots

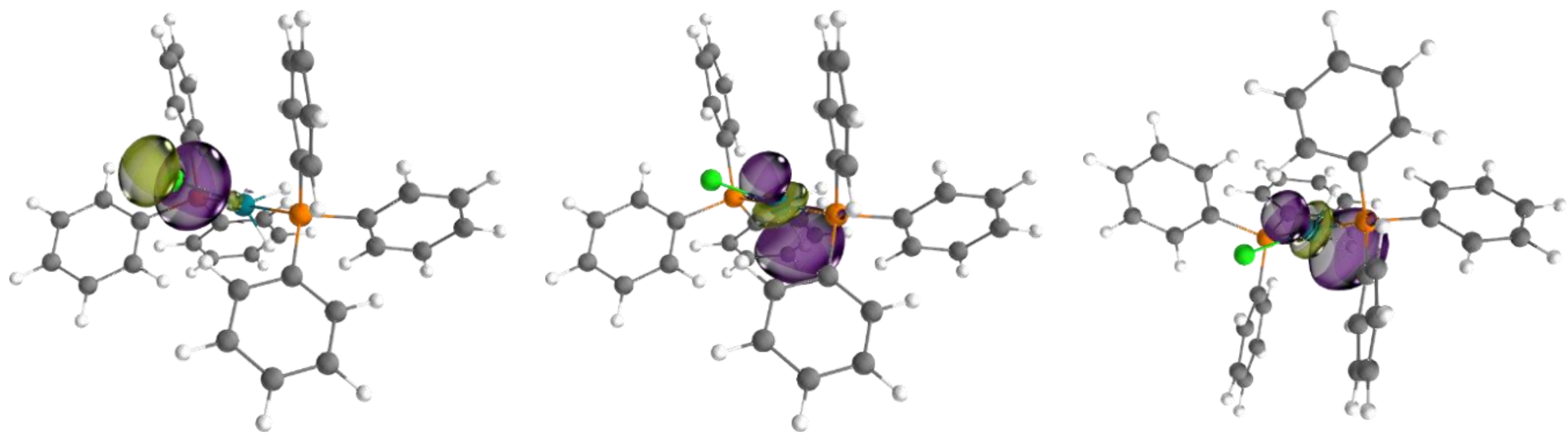

Figure SI9**.** Bonding NBOs in [$H_2Rh(PPh_3)_2Cl$] for *trans*-coordinated hydrogen atoms.
